# Fortified Anonymous Communication Protocol for Location Privacy in WSN: A Modular Approach

**DOI:** 10.3390/s150305820

**Published:** 2015-03-10

**Authors:** Abdel-Shakour Abuzneid, Tarek Sobh, Miad Faezipour, Ausif Mahmood, John James

**Affiliations:** 1 Computer Science and Engineering Department, University of Bridgeport, Bridgeport, CT 06604, USA; E-Mails: sobh@bridgeport.edu (T.S.); mfaezipo@bridgeport.edu (M.F.); Mahmood@bridgeport.edu (A.M.); 2 Department of Electrical Engineering & Computer Science, United States Military Academy, West Point, NY 10996, USA; E-Mail: john.james@usma.edu

**Keywords:** WSN, anonymity, privacy, source location privacy, sink privacy, contextual privacy, routing privacy, temporal privacy, traffic privacy, observability, safety period

## Abstract

Wireless sensor network (WSN) consists of many hosts called sensors. These sensors can sense a phenomenon (motion, temperature, humidity, average, max, min, *etc.*) and represent what they sense in a form of data. There are many applications for WSNs including object tracking and monitoring where in most of the cases these objects need protection. In these applications, data *privacy* itself might not be as important as the privacy of source location. In addition to the source location privacy, sink location privacy should also be provided. Providing an efficient end-to-end privacy solution would be a challenging task to achieve due to the open nature of the WSN. The key schemes needed for end-to-end location privacy are anonymity, observability, capture likelihood, and safety period. We extend this work to allow for countermeasures against multi-local and global adversaries. We present a network model protected against a sophisticated threat model: passive /active and local/multi-local/global attacks. This work provides a solution for end-to-end anonymity and location privacy as well. We will introduce a framework called fortified anonymous communication (FAC) protocol for WSN.

## 1. Introduction

Wireless sensor networks (WSNs) consist of many hosts called sensor nodes (SNs). A wireless sensor device is a simple autonomous host device. It can sense a phenomenon, convert the sensed information into a form of data, process the data and then transmit the data to a sink or a base-station (BS) for further usage or analysis. The sensor host is very limited in terms of storage, cache memory, processing and computing power, communication capabilities and battery lifetime [1,2,3,4]. There are many different applications adopting sensor nodes. However, this work focus on monitoring and tracking applications where sensor nodes monitor a certain area and track the presence of a certain object of interest such as an animal in the wildlife, a patient or a doctor in a hospital, or a fellow soldier or a vehicle in the battlefield. When the sensor node senses the object, it reports data to the sink (or to multiple sinks) either directly or through other neighboring sensors. One of the most common applications discussed in source location privacy (SLP) literature is the *panda monitoring game* [4,5]. When a sensor node detects a panda in a certain area, it should report via a message transmitted through intermediate nodes to the sink. In order to protect the panda from hunters or adversaries (ADVs), we need to implement in place an efficient source location privacy scheme (SLP). In such a scenario, location privacy is much more important than the confidentiality of the sensed data itself. Source location privacy is even more important in military, homeland security, and law enforcement, in addition to many other civilian applications [6]. In addition, base-station location privacy (BLP) is very crucial for every WSN since it aggregates all the data.

## 2. Problem Statement

Privacy in WSN is typically categorized into two categories: *data privacy* and *context privacy* [7,8,9,10]. The data privacy includes *data aggregation* and *data query*. The context privacy includes *routing privacy*, *identity privacy*, *location privacy* and *timing privacy.* In this work, we shall focus on using anonymity to provide *location privacy*, which includes two subcategories, source location privacy and base-station location privacy. One of the first works to classify context privacy was done by Kamat *et al.* [11,12], where they addressed the panda hunter game. They claim that the routing scheme is responsible for hiding source location of a subject. They have used two metrics to measure SLP: *Safety period* and *capture likelihood*. Safety period is the number of messages a source sends before it is captured. The capture likelihood is the probability that an adversary can capture the source within a certain period. There are generally two ways to locate a source using passive attacks: *Traffic analysis* [7,13] and *packet tracing* [7,14,15]. The traffic analysis can determine the source or sink locations by analyzing the traffic. Packet tracing can also be used to find the source location since adversaries may use radio-frequency localization techniques to perform a hop-by-hop trace. The adversary can move quickly during packet trace. It could be used to trace mobile nodes due to its fast response compared to traffic analysis [7,14]. We provide a framework that can be tested against other solutions using the following metrics: (i) *Security*: the probability that the adversary successfully identifies the source, the intermediary SNs or the sink; (ii) *energy cost*; (iii) *storage and memory cost*; (iv) *delivery time*; (v) *safety period*: how long it takes the adversary to capture the first sensor node in the network. Our proposed framework provides a modular system that could be configured for a variety of network models and for a variety of threat models. The rest of this paper is organized as follows: in Section 3, we give some background and literature survey. In Section 4, we will explain the suggested system model, network model, threat model and the traffic model. In Section 5, we will introduce the anonymity module. In Section 6, we will discuss the module of data authentication and integrity. In Section 7, we will discuss temporal privacy. In Section 8, we will have a thorough security analysis. In Section 9, we will have performance analysis and evaluation. In Section 10, we will summarize our work and suggest some additional development to the framework in the future work.

## 3. Background and Literature Survey

There are many solutions that have been presented to solve the problems of SLP and BLP. Li *et al.* [8] discussed some of the solutions for SLP but they did not aim to create a survey. The comprehensive survey for SLP was presented in the work by Conti *et al*. [4], where they categorized the solutions into eleven groups. They have discussed many solutions and compared them in terms of power consumption, the attack/threat model, view of the network, exposed information, and efficiency in providing SLP. They also discussed some issues that each solution exhibits. 

Anonymity is an old issue that was discussed for mobile networks, Ad Hoc networks and Internets. Recently, it has become a concern for WSNs. We have identified solutions for location privacy using anonymity in WSN. We have included them chronologically in Table 1.

An important solution against a global adversary introduced by Chen *et al.* [16] called efficient anonymous communication (EAC) provides sender, link and sink anonymity. We know that anonymity is not enough to achieve fortified end-to-end privacy. There are some solutions based on fake data sources where SNs send out fake packets to other nodes within the network. Some literature call them dummy packets. A fake packet does not contain any real information about any real events but it helps to obfuscate the real traffic and to divert the adversary by mimicking the presence of a fake source. The literature shows reasonable solutions using fake sources. Some of them are designed to handle a local adversary and some of them are suitable for a global adversary. Some of the literature presume a certain routing scheme, topology, network and threat models. Ouyang *et al.* [17] introduced three different solutions to handle the global adversary problem. The first solution is the globally optimal algorithm (GOA). Each SN has a pseudo random number generator that defines the interval time. The second solution by Ouyang *et al.* [17], is the heuristic greedy algorithm (HGA) where SNs follow the same procedure as in GOA except that the SN does not know the complete topology, but it only has the information of its location and the seeds of its neighbors. The third solution by Ouyang *et al.* [17] is the probabilistic algorithm (PBA) where nodes still follow the procedure of HGA, except that they do not send fake messages at the end of every interval. It uses probability *p* to decide whether to send a fake message or not. The value of *p* will reduce the communication overhead at the expense of SLP.

We can enhance SLP and BLP by having temporal privacy against the hop-by-hop trace attack or timing analysis attack [4]. There are some literature addressed this using issuing packet delay techniques. Hong *et al.* [18] introduced probabilistic reshaping (*PRESH*) to counter the adversary that uses timing analysis techniques and also introduced and upgraded PRESH to be extended probabilistic reshaping (*exPRESH*) to counter such a scenario. The SN will delay the packet in its buffer again up to *D* time. Kamat *et al.* [19] introduced rate controlled adaptive delaying (*RCAD*).

**Table 1:** Solutions for location privacy using anonymity [4]. SAS, Simple Anonymity Scheme; CAS, Cryptographic Anonymity Scheme; HIR, Hashing-Based ID Randomization; RHIR, Reverse HIR; APR, Anonymous Path Routing; ACS, Anonymous Communications Scheme; DCARPS, Destination Controlled Anonymous Routing Protocol for Sensor Nets; MAQ, Max Query Aggregation; PhID, Phantom ID; EAC, Efficient Anonymous Communication.

No.	Scheme	View of the Adversary	Anonymity Technique	Passive Attacks	Active Attacks
1	SAS & CAS [20]	Global	Pseudonyms	Eavesdropping, SN compromise, limited traffic analysis	-
2	HIR & RHIR [21]	Global	Pseudonyms	Eavesdropping, SN compromise	-
3	APR [22]	Local	Pseudonyms	Eavesdropping, hops-tracing	SN compromise
4	DCARPS & Global DCARPS [6]	Global	Pseudonyms	Eavesdropping, hops-tracing	-
5	ACS [23]	Local	Pseudonyms	Rate monitoring, time correlation, identity analysis, hops-trace	-
6	MQA [24]	Global	Aggregation	Eavesdropping, hops-tracing	Packet injection
7	PhID [4,25]	Local	Pseudonyms	Eavesdropping, traffic analysis	-
8	EAC [16]	Global	Pseudonyms	Eavesdropping, traffic analysis	DoS, SN compromise, Traffic injection

In this work, we shall enhance EAC, the efficient anonymous communicating protocol [16,26]. An extension to EAC called *Enhanced Communication Protocol for Anonymity and Location Privacy in WSN* (E^2^AC) was presented in [27]. We will call our scheme FAC, *a fortified anonymous communication protocol for WSN.* EAC does not handle the pseudonyms synchronization very well. There are many situations where the system will get unsynchronized. It also could not handle multi-colluding adversaries and lacks a mechanism for time correlation attack. Most of the other solutions do not handle global or multi-colluding adversaries. Each of the different solutions focus on certain selected attack scenarios. Our work is aimed to be comprehensive. We propose a solution against anonymity attacks, temporal attacks, transmission rate-analysis attacks, and statistical attacks, which altogether will provide a fortified source and sink location privacy.

## 4. System, Network, Threat and Traffic Models

In this work, a framework for end-to-end location privacy using anonymity and temporal privacy is presented. The framework provides the following security elements: Sender anonymity, receiver anonymity, link anonymity, SLP, BLP, data privacy, safety period, and energy preservation. We use sink and BS alternatively throughout this work. The system would be fed with inputs such as the nature of the adversaries in the network, the residual energy in the SNs, the desired lifetime or safety period. We assume bi-directional links where two nodes are considered neighbors if and only if they can hear each other [6].The network considers one sink, which collects/aggregates sensed data (stimuli) from all the SNs. The sink works as an interface for WSN to the wired network [20]. Data packets generated by SNs are ultimately destined uplink to the sink and never destined to another SN. However, it could go through a multi-hop path. Control packets can be sent from the sink, downlink, to the SNs by unicast or by broadcast messages. To enhance BLP, the sink acts like any other SN in the network while communicating with the SNs to make it absolutely indistinguishable. Most of the literature show that the operation of WSN network goes through two or more phases. However, generally speaking, the WSN runs in three phases: *Pre-deployment phase*, *setup phase*, and *communication phase.* We assume that the SNs have the ability to obfuscate the addresses at the MAC level header [20,28]. All sensors are time synchronized using *time synchronization protocol* [20].

**Figure 1:**
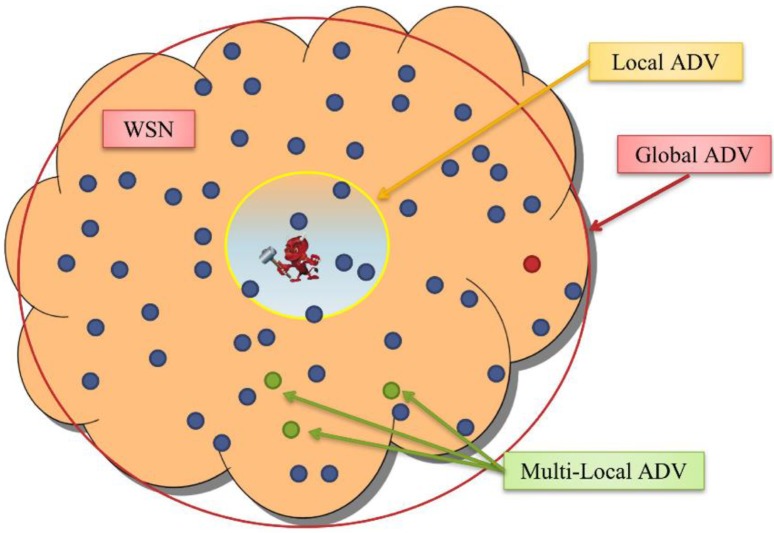
The view of adversary in WSN: local, global and multi-local.

The WSN will need a protocol for *network topology discovery* that allows the sink to view the global topology of the network without revealing the location of the sink [6]. The adversary nodes have very strong capabilities compared to the SNs. They are resource-rich; sufficient energy supply, computation/processing capabilities, and unlimited storage memory. An adversary could run both *passive* and *active* attacks. We presume *Kerchhoff’s Principle* [29] for our framework, where the adversary knows everything about the system except the *keys* and *IDs*. The framework will be able to handle both passive and active attacks. We presume that only few compromised nodes could coexist at one time due to the implementation of intrusion detection system (IDS) [16,30,31,32,33]. We assume a global adversary, which can monitor the traffic of the entire network and can determine the node responsible for the initial transmission, as in Figure 1. 

**Figure 2:**
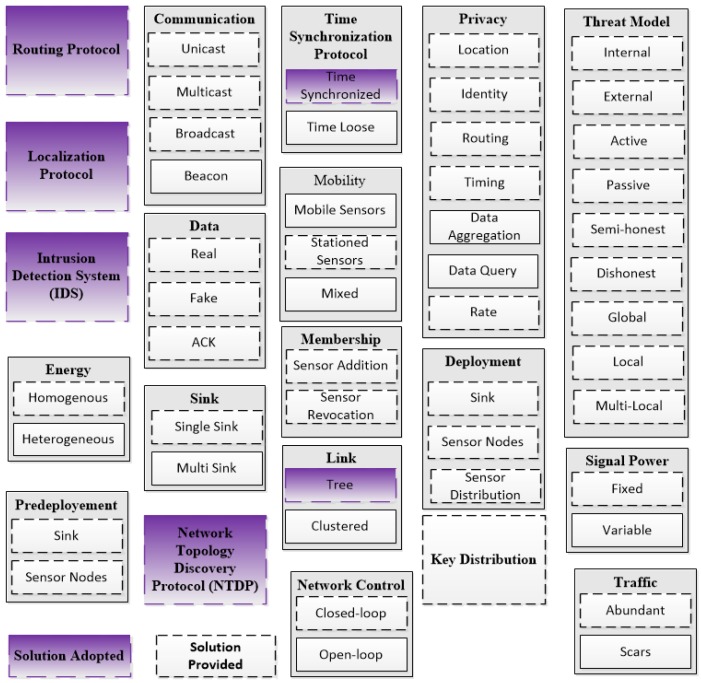
Adopted and provided modules for the framework.

Assuming a global adversary means: (a) the worst-case scenario for area coverage where colluding sensors can cooperate to cover the whole network area [34]; and (b) the worst-case scenario for timing where the coverage area of the adversary is not known to the privacy protocol at any time [34]. We also assume that the adversary is capable of observing transmissions over extended periods. It is, however, not able to break the encryption algorithms or the hash functions used for securing data during transmission. We presume *abundant* traffic where sensors detect and transmit many packets such as in the applications of environment monitoring. Such networks can resist global eavesdroppers easily comparted to *scarce* traffic networks due to the volume of transmissions that could happen at one time. The framework is built of many blocks of *functions* and *protocol*. We have adopted some of the solutions provided in the literature such as solutions for localization and time synchronization. Figure 2 provides a list of all the blocks that we have provided solutions for and the blocks that we have adopted. The BS will be able to control the network by assigning the value of different parameters.

## 5. Module I: Anonymity

The communication process is divided into three phases, namely: Pre-deployment phase, setup phase and communication phase.

### 5.1. Pre-Deployment Phase

Prior to actual distribution of the SNs in the field of application, the SNs need to be tested, fully charged, and preloaded with some parameters. We will use subcase letters i and j to describe source and intermediary nodes consecutively. We will use *BS* to describe the sink or the base station. Table 2 summarizes all the parameters and terms used in this work.

**Table 2:** Reference of important parameters and terms used by FAC.

Notation	Definition	Source
ID_i_	ID of sensor i	Preloaded
a_i_	Random number shared between SN_i_ & BS	Preloaded
b_i_	Random number shared between SN_i_ & neighbors	Preloaded
c_i_	Random number shared between SN_i_ & neighbors	Preloaded
H1	Hash function to create pseudonyms and the keys	Preloaded
H	Hash function to create data digest	Preloaded
k_i↔bs_	Pair-wise key shared between SN_i_ & BS	Preloaded
kb_i_	Broadcast key for SN_i_	Preloaded
fkb_i_	Fake broadcast key for SN_i_	Preloaded
N	Number of SNs in WSN	Learned
N_i_	Number of neighboring for SN_i_	Calculated
HC_i↔bs_	Hop-count between SN_i_ & BS	Learned
PID_i_	Pseudonym ID shared between SN_i_ & BS	Calculated
BPID_i_	Broadcast pseudonym ID	Calculated
a_i↔j_	Random value shared between SN_i_ & SN_j_	Calculated
k_i↔j_	Pair-wise key shared between SN_i_ & SN_j_	Calculated
OHPID _i↔j_	Pseudonym ID shared between SN_i_ & SN_j_	Calculated
APID_i_	ACK pseudonym ID for SN_i_	Calculated
FBPID_i_	Fake broadcast pseudonym ID	Calculated
T_i_	Table in SN_i_ for shared parameters	Calculated
TIME_STAMP	Time stamp	Calculated
SEQ_NO	Sequence number for a message	Calculated
TTL	Time to live	Calculated
MCG_LGTH	Message size	Calculated
$\Delta_{residual}$	Residual energy	Calculated
⊕	XOR Operation	Operation
||	Concatenation operation	Operation

### 5.2. Setup Phase

It is typical to presume the WSN is considered secure for some short period after the deployment of sensors and before the steady communication phase. Zhu *et al.* [35] presented that WSN has a lower bound on the time interval (T_min_) before the adversary is able to compromise a SN. During this time, the sensors can communicate and exchange all needed information safely. The sink needs to know the location of all the SNs participating in the WSN. Likewise, the SNs need to know their relative locations to the sink and to their neighbors. There are many *localization schemes* which, are proposed in the literature [16,20,36,37]. We presume the network will adopt one of the available efficient localization schemes. Localization allows each SN to know its smallest *hop-count* to the BS (*HC_i↔bs_*).

#### 5.2.1. Creating Pseudonyms

The key idea is to use pseudonyms instead of using real IDs for the SNs and the BS during communication. Therefore, one disposable pseudonym per one transmission is used. This way, the ADV cannot trace back to the source using multiple messages containing the real ID. There are five kinds of transmissions that could happen in the WSN: (i) Multi- hop transmission between SN_i_ and BS; (ii) transmission between two sensor neighbors i and j; (iii) broadcast sent by SN_i_ or BS; (iv) acknowledgement; and (v) fake broadcast. The process starts by creating a pseudonym ID for each SN_i_, we call it for short (PID*_i_*) which is computed using Equation (1):

PID_i_ = H_1_(ID_i_ ⊕ a_i_) (1)

The SN_i_ can calculate the broadcast pseudonym ID (BPID_i_) according to Equation (2):

BPID_i_ = H_1_(ID_i_ ⊕ b_i_) (2)

The SN_i_ can calculate the fake broadcast pseudonym ID (FBPID_i_) according to Equation (3):

FBPID_i_ = H_1_(ID_i_ ⊕ c_i_) (3)

SN_i_ should, by now, know its entire neighbor set (N_i_). SN_i_ will send a broadcast discovery message (M_discovery_), to exchange parameters with all one-hop neighbors. The format of the message is stated in Equation (4):

M_discovery_ = K_dis_(TTL || ID_i_ || k_i↔bs_ || kb_i_ || fkb_i_ || a_i_ || b_i_ || c_i_ || Δ_i_ || HC_i↔bs_) (4)

where TTL should be 1 for this transmission. K_dis_ is a shared common encryption key to secure the discovery message. SN_i_ will receive also a similar broadcast message from SN_j_ and from all other neighbors. Both SN_i_ and SN_j_ will calculate a new random value (a_i↔j_) according to Equation (5):

a_i↔j_ = H_1_(ID_i_ ⊕ ID_j_) (5)

Both SN_i_ and SN_j_ will calculate also a new pair-wise key k_i↔j_ according to Equation (6):

k_i↔j_ = H_1_(k_i↔bs_ ⊕ k_j↔bs_) (6)

SN_i_ also calculates broadcast pseudonym ID for SN_j_ (BPID_j_) according to expression Equation (2) since SN_i_ has already received the values of ID_j_ and b_j_ through M_discovery_. It also calculates the one-hop pseudonym ID (OHPID _i↔j_) shared between SN_i_ and SN_j_ as expressed in Equation (7):

OHPID _i↔j_ = H_1_(a_i_ ⊕ a_j_) (7)

Finally, acknowledgement pseudonym ID for SN_i_ (APID_i_) will be calculated according to Equation (8):

APID_i_ =H_1_(ID_i_) (8)

SN_i_ will create a table (T_i_) which contains the shared values with the neighbors as listed in Table 3. In conclusion, we have replaced the ID with *quintuple pseudonyms* to reference the SN during the communication.

**Table 3:** Shared values among sensor neighbors. If SN_i_ has N_i_ neighbors, then T_i_ will have N_i_ tuples.

Information in T_i_ Per Each Neighbor	Tuple for SN_j_
Shared random number	a_i↔j_
Shared broadcast random number	b_j_
Shared fake broadcast random number	c_j_
Shared broadcast key	BPID_j_
Shared fake broadcast key	FPID_j_
Shared one-hop key	k_i↔j_
Current one-hop pseudonym ID	OHPID _i↔j_
Link direction	link_i→j_
Residual energy level	Δ_j_

#### 5.2.2. Deleting Security Information

After storing all required pseudonyms, parameters and keys in T_i_, it would be the time to delete all unnecessary information from SN_i_ memory for the purpose of security [27]. In addition, it will release some memory storage space [16,26]. Most importantly, SN_i_ will delete ID_i_ and HC_i↔bs_, which could be critical information for the adversary. In addition, SN_i_ shall delete all discovery messages.

### 5.3. Communication Phase

During the communication phase, when sensing and sending data to the BS takes place, there are seven operations that continue until network lifetime ends. These operations are: (i) Sense and send a message to a neighbor; (ii) forward a message hop-by-hop; (iii) broadcast a real message; (iv) acknowledgement; (v) broadcast a fake message; (vi) SN removal; and (vii) SN addition. A SN will have three roles, in terms of data transmission, during the communication phase: (i) Role as a sensor; (ii) as a message forwarder; and (iii) as a broadcaster. In the following sections, we will use SN_i_ as a source node and SN_j_ as a neighbor to the source.

#### 5.3.1. Transmission as a Sensor

When SN_i_ senses data, it needs to send a message hop-by-hop to the BS. The SN_i_ only recognizes itself by its (PID_i_), and the BS will recognize the source of the message by its PID_i_ as well. Thus, the PID_i_ of the source needs to be included in the message until the BS receives it. Consequently, the PID of a sensor will be updated after every transmission. The SN_i_ needs to select one neighbor from N_i_ to forward the message to it. The selection process goes through a probabilistic protocol, which guarantees that SN_i_ does not use one neighbor all the time when forwarding its data; first, for routing privacy, and second for increasing the lifetime of the WSN. SN_i_ will form the message in the following format:

M_i→j_ = OHPID_i↔j_ || E_ki→j_ (PID_i_ || E_ki↔bs_ (D_i_)) (9)

where D_i_ includes the sensed data. Once SN_i_ knows that the message (M_i→j_) is delivered to the the neighbor, it needs to dispose of the current pseudonym PID_i_ and issue a new one for the next transmission as indicated in Equation (10):

PID_i_ = H_1_(PID_i_ ⊕ a_i_) (10)

In addition, both SN_i_ and SN_j_ will dispose of the current OHPID_i→j_ and issue a new one for the next communication between the two neighbors according to Equation (11):

OHPID_i→j_ = H_1_(OHPID_i→j_ ⊕ a_i→j_) (11)

The message (M) will then be reformatted by the recipient SN_j_ and again forwarded to the next node, say SN_r_, and so on, until it gets to the BS. If SN_j_ was the BS, then the BS uses the shared one-hop key between the sensor and the BS, to decrypt the data and to get the PID_i_, which the BS can use to recognize the source SN_i_. Only at this point of time, BS can update the value of PID_i_ of SN_i_. It also reads the data (D_i_) which the BS can decrypt using k_i↔bs_.

#### 5.3.2. Transmission as a Forwarder

When SN_i_ sends the message one-hop uplink to the neighbor SN_j_, then SN_j_ needs to forward the message to another intermediary node. Upon receiving M_i→j_, SN_j_ will match OHPID_i→j_ in its table, T_j_. If there is no match, then the message definitely is not addressed for SN_j_ and it will be dropped immediately. If it matches, then the message is decrypted using k_i→j_. The message will be forwarded to SN_r_ after (M) is reformatted as in Equation (12):

M_j→r_ = OHPID_j↔r_ || E_kj→r_ (PID_i_ || E_ki↔bs_ (D_i_)) (12)

**Figure 3:**
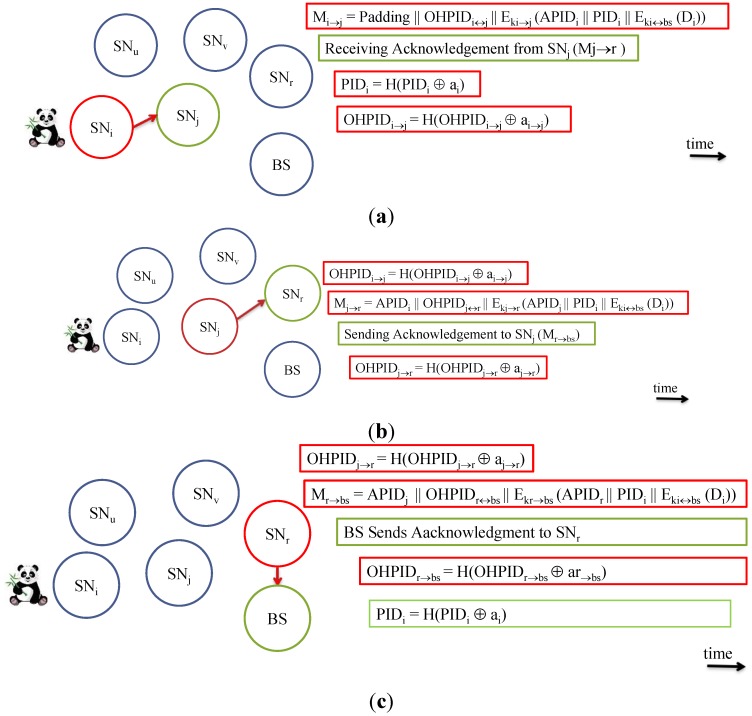
The sequence of a message transmission from SN_i_ to the BS. (**a**) SN_j_ receives a message from SN_i_; (**b**) SN_j_ forwards the message to a neighbor SN_r_; (**c**) BS receives the message and processes it.

Right after the data is *received* by SN_j_ and *forwarded* to the next one-hop SN_r_, the SN_j_ updates the pseudonym OHPID_i↔j_. SN_j_ now is ready to exchange another message with SN_i_ using the new pseudonym OHPID_i↔j_. However, SN_j_ is not yet ready to send data to SN_r_ since SN_r_ does not update the OHPID_j↔r_ until (D_i_) is forwarded to the next hop, say NS_v_. See Figure 3 for the sequence of transmissions for a message from SN_i_ to the BS.

#### 5.3.3. Acknowledgement

As expected in data networks, message could be lost or could be corrupted. In either case, retransmission is required. Because SNs change PIDs after each transmission, synchronizing PIDs is crucial. Updating the pseudonyms depends on successful message delivery. Ideally, the source should update the pseudonyms only after making sure the BS receives the data. However, the lack of direct connection between the source and the BS makes it a bit complicated process.

The BS cannot send direct acknowledgement to the source if it is multiple hops away. We have to depend on multiple acknowledgements along the path between the source and the BS. SN_i_ needs to calculate the Acknowledgement pseudonym ID (APID_i_) according to Equation (13):

APID_i_ = H_1_(APID_i_ ⊕ b_i_) (13)

The message will be sent out to the neighbor with the current value for APID_i_. Thus, we will rewrite M_i→j_ as it appears in Equation (14):

M_i→j_ = Padding || OHPID_i↔j_ || E_ki→j_ (APID_i_ || PID_i_ || E_ki↔bs_ (D_i_)) (14)

Padding is added to make sure all the one-hop messages have the same size to prevent *size correlation* attacks. When SN_j_ receives the message, it will reformat the message as in expression Equation (15) and then send it to SN_r_:

M_j→r_ = APID_i_ || OHPID_j↔r_ || E_j→r_ (APID_j_ || PID_i_ || E_ki↔bs_ (D_i_)) (15)

**Figure 4:**
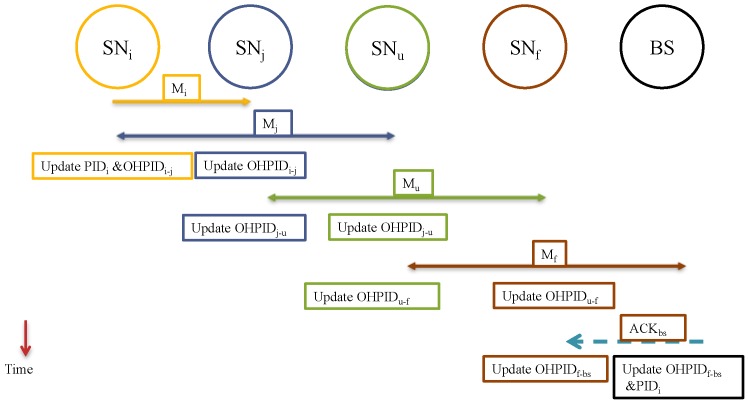
Using APID_i_ for acknowledgement with no errors.

**Figure 5:**
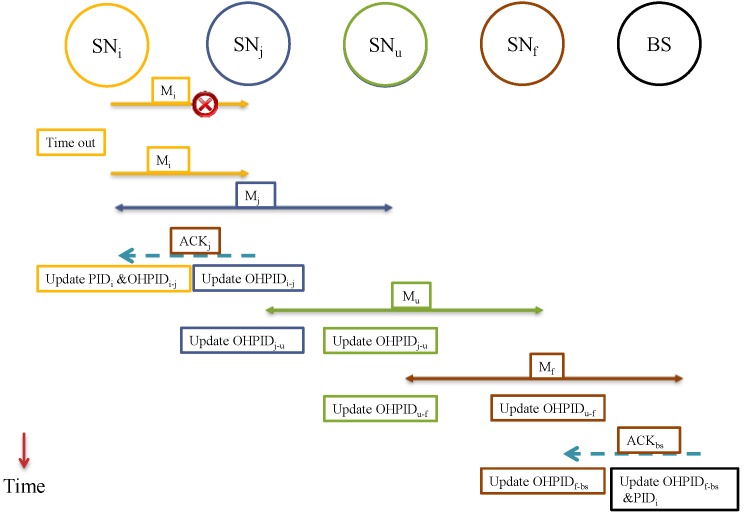
Acknowledgement for a message with errors.

The transmission of M_j→r_ should be heard by all the neighbors including both SN_i_ and SN_r_. If SN_i_ hears the message and reads (APID_i_), the SN_i_ knows that M_i→j_ was received correctly by SN_j_. Only at this time, SN_i_ updates the value of OHPID_i↔j_. PID_i_ will get updated, as well, since SN_i_ is the source of the message. This is exhibited in Figure 4. Here are two scenarios:

*Scenario 1:* The packet sent by SN_i_ is lost or got corrupted. In this case, SN_j_ considers nothing happened, so it will not forward any message onward. Meanwhile, SN_i_ will wait for (*ζ*) time to expire. It will send the message again with updated APID_i_. Once the message is acknowledged according to the procedure explained earlier, then PID_i_, OHPID_i_ and APID_i_ will be updated. If it is intermediary SN, only OHPID_i_ and APID_i_ is updated as exhibited in Figure 5.

*Scenario 2:* SNj receives the packet correctly; the new packet M_j→r_ is sent out which contains the acknowledgement (APID_i_), and SN_j_ updated the value of OHPID_i↔j_. However, SN_i_ does not hear the forwarded message M_j→r_ within time (*ζ*). At this moment SN_i_ does not know for sure if the message was delivered (*resembles scenario 1*), or the acknowledgement is lost. It has to account for the worst case. A copy of the message will be retransmitted to SN_j_ with the current OHPID_i_ and updated APID_i_. SN_j_ can recognize the message because of the value of old OHPID_i_. After receiving the *retransmitted* message, it now sends a direct acknowledgement to SN_i_ as in Equation (16).

ACK_i←j_ = APID_i_|| Padding (16)

**Figure 6:**
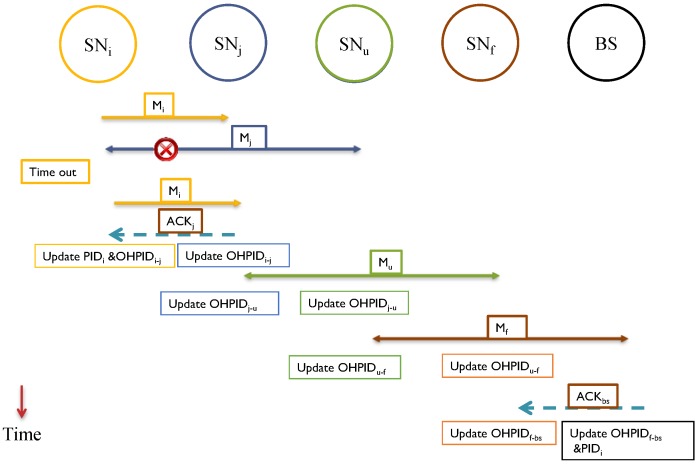
Handling lost acknowledgement.

Figure 6 shows the process. BS is treated similar to a normal SN, so it has to acknowledge every message it receives. After the message is delivered to the BS, and after the message is acknowledged, the PID_i_ (of the source) will be updated on the BS tables while it has been already updated in the sensor itself after the first acknowledgement.

**Figure 7:**
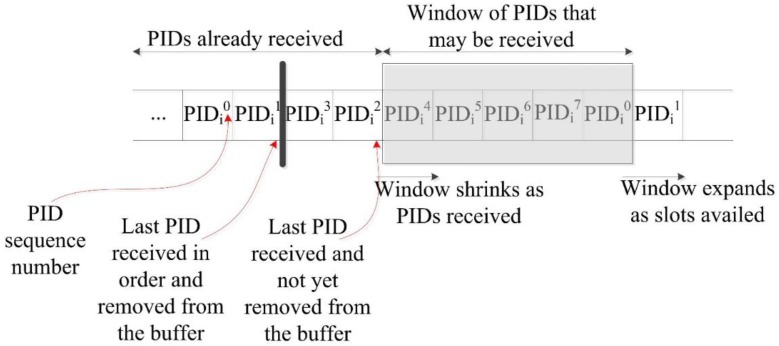
Sliding window for received PIDs [27].

Both the SN_i_ and the BS will be ready to exchange a new message. As long the new message does not reach to the BS before the old PID_i_ is updated, the system will remain synchronized. This way, we have a possible window of one message only. We propose implementing a *sliding window* mechanism as exhibited in Figure 7 [27]. For each sensor, we can have a window of (W) slots.

#### 5.3.4. Transmission as a Broadcaster

Typically, the BS is required to broadcast a message for control and management purposes. Likewise, a sensor might need to broadcast a message to the BS or to the neighbors for network setup, maintenance and other management issues. The framework requires keeping all the messages indistinguishable throughout the network, so all the messages need to have the same size. Each SN is preloaded with a broadcast key (kb_i_) and assigned broadcast pseudonym (BPID_i_). The broadcast message sent by SN_i_ is formatted as in Equation (17):

M_b_ = Padding || BPID_i_ || E_kbi_(D_b_) (17)

All the neighbors will receive the broadcast message from a source SNi. SN_i_ and the recipients will update BPID_i_ according to Equation (18).

BPID_i_ = H_1_(BPID_i_ ⊕ b_i_) (18)

Upon receiving the broadcast message (M_b_), SN_j_ decrypts the message using (kb_i_) stored in the table (T_j_). It then encrypts it again using (kb_j_) and broadcasts (M_b_) to its one-hop neighbors set (N_j_) as in Equation (19):

M_b_ = BPID_i_ || BPID_j_ || Ekb_j_(D_b_) (19)

When the BS receives a broadcast message, it is ultimately the destination, so intuitively it does not need to broadcast the message again. Our proposed framework assumes that the BS behaves similar to a normal sensor. To maintain this pre-course, we require the BS to broadcast the message again for acknowledgement purpose. Thus, we introduce the limited broadcast where the BS will be able to broadcast to only one hop (TTL = 1).

#### 5.3.5. Limited Broadcast Messages

A sensor inside network maze can only recognize the neighboring sensors and the BS. When SN broadcasts a message uplink (towards the BS), then all neighbors should hear it. The neighbor should broadcast the message again if and only if the message comes from a SN with a bigger hop-count (HC). This will conserve a lot of unnecessary traffic and energy dissipation. The broadcast message will contain (TTL = HC). The value will keep decreasing by one until it gets to the BS. In contrast, the downlink broadcast messages (by the BS to the SNs) should have (TTL = 0) where the intermediary sensors would rebroadcast the message if and only if it comes from a neighbor with a smaller (HC). A special case when (TTL = 1) where the message will be broadcasted to one-hop neighbors only. FAC also may adopted a more sophisticated optimized flooding algorithm for wireless multi-hop network, such as CDS-based algorithms [38,39].

#### 5.3.6. Fake Broadcast Message

The sensors need to send fake messages to prevent *time correlation*, *rate analysis* and *statistical analysis*. A fake message is technically a one-hop broadcast message. However, to prevent correlation, the message needs to behave similar to real messages. Therefore, the message needs to be encrypted and have similar size as the real message to make it completely *indistinguishable*. Since it has to carry a dummy data, it will contain the *residual energy* (Δ) of the issuing sensor. This information will be extracted by the recipient neighbors and saved in the related tuple in the table (T). The fake broadcast message sent by SN_i_ is as in Equation (20):

M_f_ = Padding || FPID_i_ || E_kfi_(Δ_i_) (20)

All the neighbors will receive the fake broadcast message from SNi. SN_i_ and the recipients will then update FPID_i_ according to Equation (21):

FPID_i_ = H_1_(FPID_i_ ⊕ c_i_) (21)

There is no need to worry about the pseudonyms synchronization since the main purpose of the fake messages is to show activity in idle sensors to obfuscate real messages.

### 5.4. SN Removal

There are many reasons why a sensor should be removed from WSN. For instance, when the battery of a sensor is about to deplete, it should refrain from participation. This would protect against data loss and maintain the pseudonyms synchronized. In some other cases, WSN use IDS [40,41] to protect against active attacks, so once a sensor is captured, it must be banished from the network. Procedurally, if SN_i_ opts to be removed, it will send a message to the BS as in Equation (22):

M_i→j_ = OHPID_i↔j_ || E_ki→j_ (PID_i_ || E_ki↔bs_ (D_remove_)) (22)

where (D_remove_) is a command to banish the sensor. The tuple of the SN_i_ in the BS tables will be disabled permanently. In addition, SN_i_ will send a broadcast message to the neighbors as in Equation (23):

M_b_ = Padding || BPID_i_ || E_kbi_(D_remove_) (23)

Once the neighbors get the message (D_remove_), they will delete the tuple related to SN_i_ from the table (T) and banish the sensor. The BS for sensor removal could use the same process.

### 5.5. SN Addition

To add a new sensor to the network, the sensor will be preloaded with the required parameters: ID_i_, a_i_, b_i_, c_i_, H1, k_i↔bs_ and kb_i_, and fkb_i_. Right after deployment, the sensor calculates the shared parameters with its neighbors. The BS should be trusted to run the process. The BS will send special key (*k_add_*) to all the neighbors. SN_i_ will be preloaded with the same key as well. SN_i_ and the neighbors will use this special key to authenticate with each other. Initially, the BS sends the following message to the one-hop neighbors of the new sensor as in Equation (24):

M_b_ = Padding || BPID_bs_ || E_kb-bs_(D_add_) (24)

where (D_add_) is expressed in Equation (25):

D_add_ = hc || k_add_ (25)

The initial value for hc is *zero*. It will be incremented every time the message is forwarded.

### 5.6. Contribution of Anonymity Module

Other works have provided anonymity using pseudonyms and aggregation to provide SN anonymity while very few provided BS anonymity. Our anonymity module has contributed with an innovative approach by using 100% anonymous communication. We have provided to have anonymous real, fake, acknowledgment, unicast and broadcast message transmission. Moreover, we have provided anonymous transmission for the BS by providing limited onion encryption. Compromising a SN in some other works would lead to the discovery of the pseudonyms, which are, related the SN, which could help the adversary to carry further attacks. In our module, capturing a SN will not lead to pseudonyms’ leakage. The module will fight against local, multi-local and global adversary. Although, some solutions claimed fighting global anonymity, keeping the pseudonyms synchronized was not possible. We have provided a complete mechanism for synchronization, secure sensor addition and removal. The module will fight both passive and active attacks. A complete anonymity and security analysis is be provided in Section 8. Section 9, explains how the solution remains light compared to the other works.

## 6. Module II: Data Authentication and Integrity

The data is encrypted before transmission to protect against passive attacks such as eavesdropping. For active attacks, such as data and transaction falsification, message authentication is required. The two important security aspects to achieve: (i) Verify that the content of the message is not altered and; (ii) the source is authentic. We could achieve authentication by either using a message authentication code (*MAC*), or one-way hash function (*OWH*). MAC would require the sender (SN_i_) and receiver (BS) to share a secret key. The authentication code is calculated as *MAC = F (k, D)*. *DES* or other algorithms can be used to generate the code. The OWH also accepts a variable size message (*D*) as input and produces a fixed sized digest *MD = H (D)* as output. Examples for OWH are *SHA*, *MD5*, *Whirlpool and* HMAC. The advantage of OWH over MAC is the fact that it does not use encryption, which is quite slow. Comes in the middle, HMAC which is a MAC derived from OWH such as *SHA-1*. It could be expressed as: MD *=* HMAC (K, D).

If we opt to use HMAC as an example, the (M_i→j_) will be rewritten as in Equation (26):

M_i→j_ = APID_i_ || OHPID_i↔j_ || E_ki→j_ (APID_i_ || PID_i_ || E_ki↔bs_ (Di)) || **HMAC_ki↔bs_** (**PID_i_** || **D_i_**) (26)

The key (k_i↔bs_) is shared between SN_i_ and the BS. The message could be authenticated with MD using OWH as in Equation (27):

M_i→j_ = APID_i_ || OHPID_i↔j_ || E_ki→j_ (APID_i_ || PID_i_ || E_ki↔bs_ (D_i_) || **H(PID_i_** || **E_ki↔bs_** (**D_i_**))) (27)

As it is transparent from expression Equation (27), we need more processing time and therefore more power consumption because we have encrypted a sizable packet. There is a tradeoff between higher security and energy conservation. The first approach is more appropriate. Authentication for the broadcast messages is done as in Equation (28):

M_b_ = Padding || BPID_i_ || E_kbi_(D_b_) || HMAC_kbi_(D_b_)) (28)

Alternatively, it can be achieved using Equation (29):

M_b_ = Padding || BPID_i_ || E_kbi_(D_b_ || H(kb_i_ || D_b_)) (29)

The message could contain other important information such as *sequence number* (similar to the well-known *HDLC* and *TCP* protocols) and *time stamp*. The receiver uses the sequence number to verify the order of messages. Time stamp is used to check the delay threshold. Both checks will enhance protection against various active attacks. The message core data (D_i_) could have the following format:

D_i_ = SEQ_NO || TIME_STAMP || MSG_LGTH || SENSED_DATA (30)

Providing authentication to protect against active attacks is crucial in any communication. The innovation of our authentication module is by providing message authentication for every transmission in the network without limiting it to real messages unlike many other works proposed. The adversary can utilize any captured transmission to launch attacks against the network, which could include real, fake and acknowledgement messages. Our module can use MAC, OWHF and HMAC according to the security needs of the WSN. The network can adjust the parameters according to the security situation using adaptive framework. Integrating the authentication module with the anonymity module without hindering the performance of either one is a necessity, which we have achieved in this work.

## 7. Module III: Temporal Privacy

**Figure 8:**
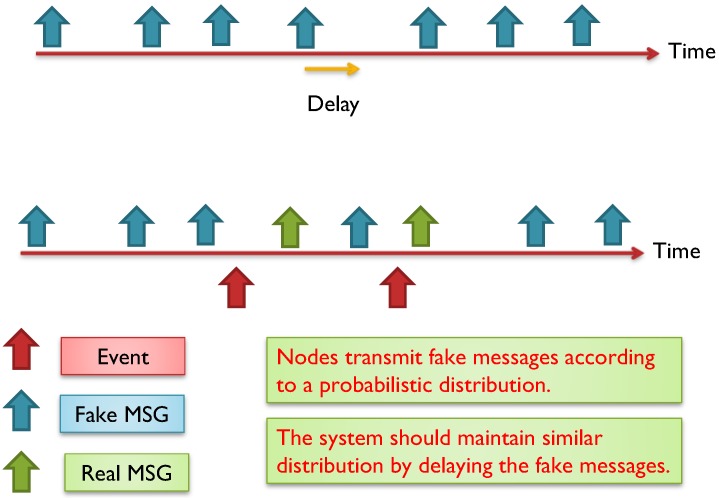
Nodes transmit fake messages according to a probabilistic distribution. When real messages are sent, the system should maintain the required distribution by delaying some fake messages [34,44].

WSN could suffer from time correlation attacks [11,13,14,23,42] by observing the time between correlative packets sent and received in a certain neighborhood. The adversary can trace forward and backward the messages until they reach to the BS or to the source. Hence, hiding temporal information is crucial for both anonymity and location privacy. Using routing schemes to protect against time correlation attacks is found to be efficient to certain extent where local adversary usually has limited mobility and partial view of the network traffic. However, routing based schemes do not work for global adversary where the traffic of the whole network can be easily monitored with a full spatial view and the adversaries can collude together to promptly detect the origin and time information of the event [18,34]. A mechanism is required to divert attention of the adversary when there is event-driven transmissions, especially with the presence of global adversary [43]. Figure 8 exhibits a probabilistic distribution for the fake messages.

The distribution of events changes which could be a reason for the adversary to detect the event timing and thereafter the source of the event. The message distribution (both real and fake) needs to be adjusted to prevent time correlation. In some applications, such as monitoring and surveillance, we cannot guarantee a certain event distribution. The literature talk about three ways to maintain an obfuscated message distribution: (i) By issuing message delays; and (ii) by issuing fake messages; and (ii) by using both delays and fake messages. Using delays works well against local adversary but might not be suitable for time sensitive networks. In contrast, using fake messages is required to protect against multi-local and global adversary, however, it is very expensive in terms of energy dissipation. Furthermore, adversary with good statistical analysis can easily detect the message distribution if the scheme is not designed carefully [34]. Some work in the literature clearly differentiates between two terms: the *event* (of transmission) and the *interval* (of transmission). If every interval has only one transmission, then event and interval are the same, however, this might not be the case when we have multiple transmissions during one interval. So, the anonymity level depends on the capability of the adversary to distinguish between real and fake transmissions. This means, given multiple transmissions by a SN, the adversary must be unable to distinguish, with significant confidence, between transmissions carry real data and transmissions carry fake data. Alomair *et al.* [34] suggested that transmission “indistinguishability” is not enough. They claim that indistinguishability is achieved when adversary monitoring the network over multiple time intervals, in which some intervals contain real event transmissions and others do not, is unable to determine, with significant confidence, which of the intervals contain the real traffic. If intervals are indistinguishable, the individual transmissions within the interval should also be indistinguishable.

We should have a mechanism to quantify anonymity while it is used, in the literature, in different ways. However, in our work, anonymity means how to prevent the adversary from knowing the source of the message. In other words, the adversary could know that a particular sensor sent a message at one time, but it should not know that sensor is the source of the message. By delaying the real messages and by issuing multiple messages at one interval would mislead the adversary. As an example, for one transmission and one adversary, where the adversary can guess either the message is real or fake without any anonymity measurement taken, it should be 0.5 (either fake or real). Let us presume ѱ donates one adversary strategy for breaching the anonymity of the system among a set of strategies. Let us presume P_r_ is the probability that the adversary succeeds using strategyψ. The anonymity A as defined in [34] with the existence of a strategyψ, is presented in Equation (31):

A_ψ_: = 1 – P_r_, where 0 ≤ P_r_ ≤ 1 (31)

If we presume that Σ represents all possible strategies for the adversary to breach the anonymity of the WSN, the accumulated anonymity will be as in Equation (32):

A: = min (A_ψ_), where ψ ∈ Σ (32)

**Figure 9:**
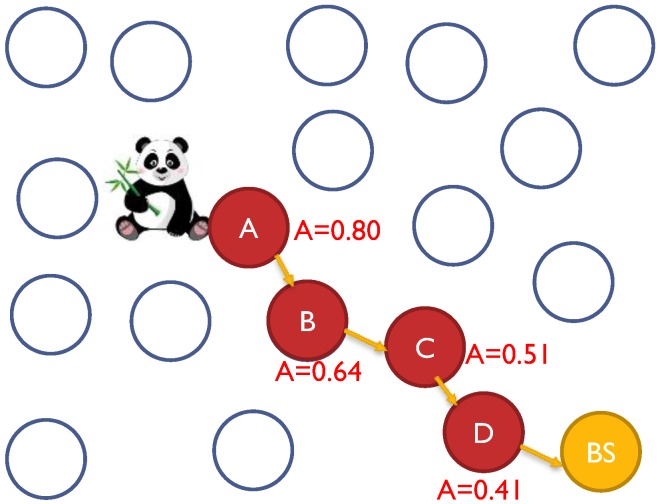
Having multiple colluding nodes will reduce system anonymity exponentially [34,42,44].

It is very important to increase anonymity for every individual SN in the network especially with the presence of multi-local or global adversaries. Presence of colluding adversaries could cause the anonymity to drop exponentially [34]. Take Figure 9 as an example, where WSN has a moving Panda from point “a”, to “b”, to “c”, then finally to “d” where each location has a SN to report the Panda’s movement. If the anonymity of each sensor is A = 0.8, then the anonymity at node “b” is A = 0.8^2^ = 0.64 and at point “d” is A = 0.8^4^ = 0.41. Having global adversary makes it super necessary to design a strong anonymity model which can resist the time correlation attack [42].

In this work, we assume the worst case for time correlation attacks which is a global or laptop-class adversary attacks [17]. Having an anonymity scheme to protect against the global adversary will be very expensive solution in terms of energy preservation and thus the lifetime of the network. In the following two subsections, we propose two schemes, the simple global anti temporal (SGAT) and the energy controlled anti temporal (ECAT).

### 7.1. Simple Global Anti Temporal Scheme (SGAT)

When an event-driven message is sent out, the adversary can trace back the message to the SN or forward to the BS. Sending few other transmissions in the network within the range of the adversary confuses it and prevents the adversary from having known path to follow. In this work, we presume the lifetime of the network (Ω) is divided into a number of intervals (*I*) and each interval time is (ω), where:

Ω = I × ω_*i*_ (33)

The value of Ω can be predicted as a range between a minimum value (worst case) Ω*_min_* and a maximum value (best case) Ω*_max_*. It all depends on how real/fake transmissions are facilitated. The SNs will send either a fake or a real message during one interval. The message is sent at the end of each interval or it is adjusted to be sent during the interval to create some variable delays through the route to the BS, which would confuse the adversary more and would prevent it from gaining useful knowledge about the network based on time correlation. SN_i_ that has sensed the event or received the real data from another SN_j_, will send the real message (*M_r_*) through a hop-by-hop path to the BS, and some other nodes will send fake messages (*M_f_*) during the same time to disrupt the adversary. There are two questions: *How long the message will be held in the SN after it is sensed?* Simplistically, M_r_ and M_f_ are sent at the end of the interval *I*. The time from arrival of the data to the end of the period time (τ_w_) expressed in Equation (34):

τ_w_ = ω_i_ − t_a_ where: t_0_ ≤ t_a_ ≤ t_s_ ≤ ω_i_ (34)

where: t_0_ is the beginning of the interval I_i_, t_a_ is the arrival time, t_0 ≤_ t_a_.

**Figure 10:**
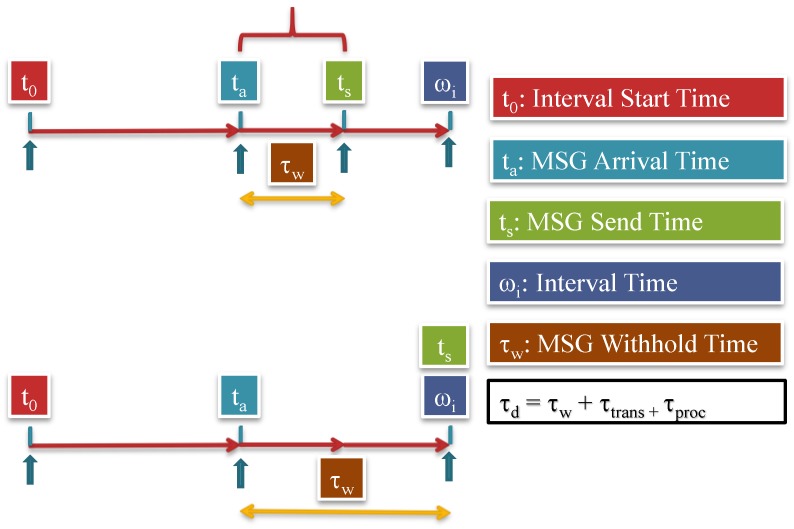
Timing for receiving a real message and then sending it out during the interval period assigned to the sensor node. The total delay will include the processing time, transmission time and the withhold time [42].

Ideally, the message will be sent immediately after it is sensed or received which makes τ_w_ = 0. Theoretically, τ_w_ could be a value: 0 ≤ τ _w_ ≤ω_i_ as exhibited in Figure 10.

*How many SNs in the network will send fake message during one interval time and which ones?* Simplistically, every SN in the network, which is in the range of the adversary and in the range of source SN, should send a fake message while SNs that have real messages will send the real messages only.

There are many technical issues regarding the determination of the optimal configuration for both questions mentioned earlier. For instance, it is not possible for the neighboring nodes to know in advance when a SN is going to sense an event. It is a completely unpredictable random-distribution for the events. The need to transmit fake messages becomes even much more crucial if we do not have busy-network. Therefore, all SNs with no real messages need to send fake messages during the interval *I_i_*, in the worst case, or only selected nodes according to a probabilistic protocol. Having high number of fake message transmissions will reduce the lifetime of the network in favor of privacy. Doing the reverse will jeopardize the privacy of the sensor nodes. The adversary could learn the mechanism of sending real and fake messages at the end of the interval. However, it is not very dangerous if the network sends enough fake messages at the same time. Having variable withhold time (*τ_w_*) is useful for privacy and for reducing the average network delay. The delivery time (*τ_d_*) presuming that the message is always sent at the end of the interval *I_i_* is:

τ_d_ = τ_w_ + τ_trans_ + τ_proc_ (35)

where: τ_d_ is delivery time, τ_trans_ is transmission time, τ_proc_ is processing time.

If we presume τ _proc_ is much smaller than τ_trans_, then τ_d_ can be rewritten as the following:

τ_d_ = τ_w_ + τ_trans_ (36)

If the message needs to go through (*U*) hops to the BS, and if we assume that the transmission only happens at the end of the Interval *I_i_*, then *t_s_*, equals to *ω_i_*, and the total delivery time (*τ_d-total_*) can be calculated according to the expression below [42]:

(37) $$\tau_{d-total}=\;\overset{U}{\underset{u=1}{\sum }}\tau_{w_{u}}+\tau_{trans_{u}}$$

Having *t_s_* equals to *ω_i_*; *i.e.*, sending message at the end of the interval, will increase the delay of the delivery presuming that every *τ_trans_* is equal [42]. Thus, optimizing *τ_d-total_* is a function of *τ_w_* according to Equation (38):

(38) $${\text{τ}}_{d-total}=\text{f}(\tau_{w})=\;\overset{U}{\underset{u=1}{\sum }}\tau_{w_{u}}$$

Each SN will be informed during the setup phase about ω*_i_* for the lifetime of the network. The BS also can alter this value by broadcast when the conditions of the WSN changes (closed-loop control). The value of ω_i_ should be calculated to achieve at least the minimum expected lifetime span Ω_min_ without jeopardizing the privacy and data integrity. Thus:

Ω_high-th_ ≥ Ω_i_ ≥ Ω_low-th_ (39)

where Ω_high-th_ is the highest possible value for Ω_i_ and, Ω_low-th_ is the lowest expected value for Ω_i_. When SN does not have a real message to send before the end of the interval period *I_i_*, it will send a fake message according to the procedure explained in the anonymity module. When SN has a real message, it will send it to one node from the neighborhood set (N_i_).

#### 7.1.1. Security Analysis

The adversary sees every SN sending a message at a fixed data rate at any one time. It also cannot distinguish any message from the rest of the messages in the network since none has similar ID. If we have N nodes in the WSN, the probability that one adversary can locate the sending node is:

(40) $$P_{r}=\;\frac{1}{N}$$

We can calculate anonymity as:

(41) $$A=1-P_{r}$$

#### 7.1.2. Delivery Time

**Figure 11:**
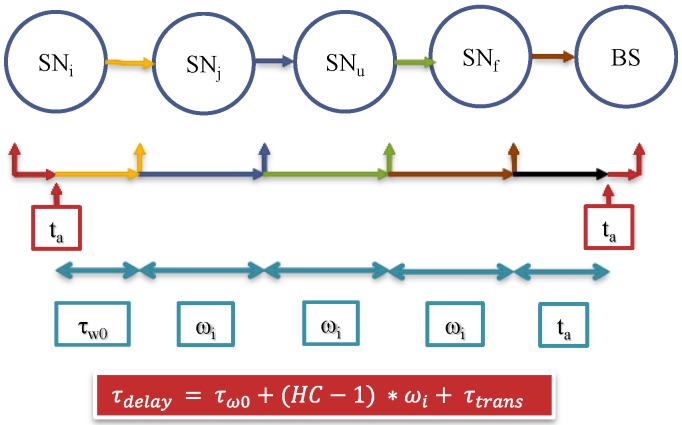
Total delay required to send a message from source to the BS through (*U*) hopes [42].

Message follows hop-by-hop path until it gets to the BS as exhibited in Figure 11. In this scheme, the message waits until the end of the interval. The delay will be calculated according to the expression below [42]:

(42) $${\text{τ}}_{d-total}=\tau_{w_{0}}+(\text{HC}-1)\text{*}\omega_{i}+\tau_{trans_{u}}$$

It axiomatic that most delay accumulates from holding the message until the end of the interval periods.

#### 7.1.3. Energy Cost

We presume in this scheme that every node would send one message at the end of each interval. The message could be either real or fake. If we have (N) nodes in the WSN, then we expect (N) messages during each interval *I_i_*. The energy spent for transmission is almost constant since we have fixed size messages. However, we can evaluate how expensive it would be to use fake messages for privacy enhancement. If we have (Q) percent of the nodes send real messages at each interval, then we are wasting (1-Q) percent of the energy and of the bandwidth.

**Figure 12:**
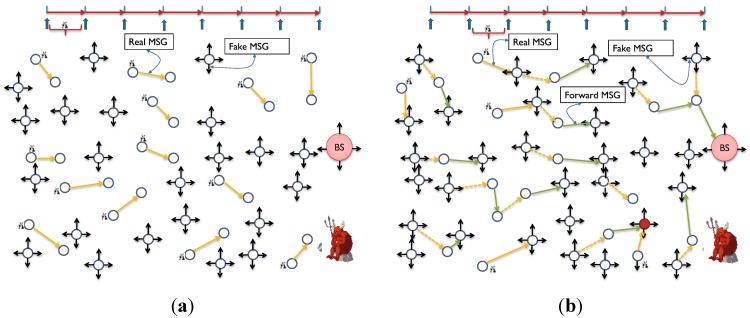
Anonymity with fake messages. (**a**) Sensors sense events and send real messages while the rest send fake messages. With Fake MSG’s: $P_{r}=\frac{1}{N}=\frac{1}{48}=2\%$, Without Fake MSG’s: $P_{r}=\frac{1}{n}=\frac{1}{11}=9\%$; (**b**) Sensors send or forward real messages will not send fake messages. The more the network gets busy the less fake messages are transmitted.

We can adjust the amount of energy consumed by increasing the interval period ω_i._ However, increasing ω_i_, would increase the delays. If a SN receives multiple messages in one interval, then it will *queue* the messages for transmission. Because the SN needs to wait until the end of the interval, it could arrange the messages in a queue and send them randomly at the end of interval. This should also increase the privacy and security of the data. It could also select a different forward node for each message. In conclusion, SGAT is energy-expensive due to sending fake/real messages by every node per each interval of time. However, SGAT provides the maximum message *entropy*. Figure 12 exhibits the network transmissions for two consecutive intervals.

### 7.2. Energy Controlled Anti Temporal Scheme (ECAT)

There are three major drawbacks in *SGAT*: (i) Having fixed interval time ω_i_ while it is possible to adjust the value for a better traffic and energy control; (ii) not considering the residual energy as a metric for selecting the forward hop; (iii) high rate of traffic due to fake messages.

#### 7.2.1. Changing ω_i_ from Fixed to Variable

Having a fixed interval time, ω_i_ could be a glitch for network performance. If ω_i_ is set to be a large value, then the delay will be high which could be a serious problem in some time sensitive applications. If ω_i_ is set to be a small value, then a huge amount of fake messages will be sent at the end of each interval, which will reduce the lifetime of SNs and accordingly the lifetime of the WSN. We propose that we have variable ω_i_ as presented in [17]. Every node will be calculating its ω_i_ using a pseudo-random number generator (PRNG). We suggest a uniform distribution algorithm such as multiplicative congruential algorithm [45,46], which is the basis for many of the random number generators in use today. Lehmer’s generators [47] involve three integer parameters, *r*, *s*, and *m*, and an initial value, *x_0_*, called the seed. A sequence is generated by the following modified formula:

X_k+1_ = b × ((r·X_k_ + s) mod m + f) (43)

**Figure 13:**
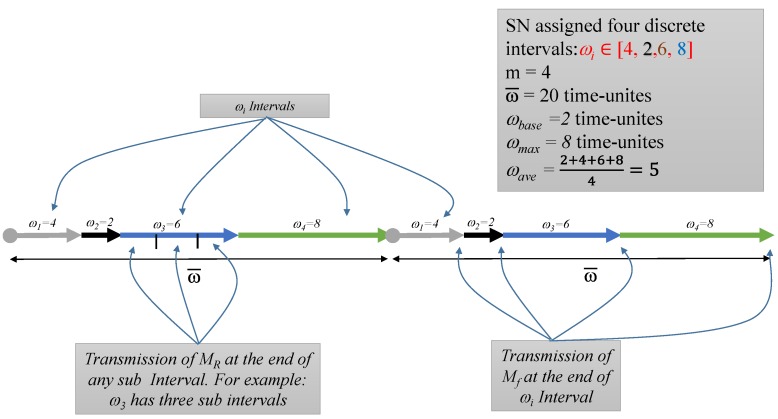
The generation of the sequence of intervals assigned for each sensor. The sequence keeps repeating for the sensor. Fake messages transmitted only at the end of the interval. However, real message could be transmitted at any subinterval.

The result of the modified PRNG will be a sequence of integer values between (b × f) and (b × (m + f − 1)). Each SN needs to be preloaded with the seed ×0, r, s, m, b and f values. The seed range is 0 to (m − 1) and it is uniformly assigned to the sensor nodes. If b = 2, f = 1 and m = 4 then sequence of four intervals will be: ω_i_ ϵ [2,4,6,8] time-units as exhibited in Figure 13. We could have up to (m!) different sequences that are uniformly distributed on the SNs. For instance, we can have Equation (24) different sequences for our example and if we have Equation (48) nodes in the network, so each sequence should be provided to two nodes only.

Each node will be dynamically assigned an interval value, which needs to change after each transmission. Taking the example above, the first $\frac{1}{m}$ th (more or less) of the sensors will send data after 2 time-units. Then, the second $\frac{1}{m}$ th will transmit after 4 time-units, and so on. At any point of time, the adversary will be faced by enough transmissions in the network that it could divert its attention far away from the SNs sending real data. By having (m) interval values where each SN will be generating one ω*_i_* using the PRNG, we have reduced the average interval time from ω_max_ to ω_ave_. That is explained in the inequality Equation (44):

(44) $${\text{ω}}_{max}>{\text{ω}}_{ave}=\frac{T_{1}+\ldots +T_{m}}{m}>{\text{ω}}_{min}$$

**Figure 14:**
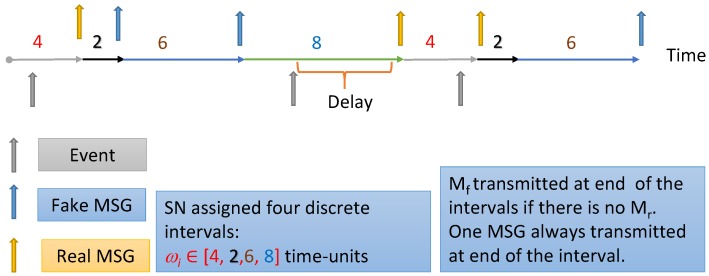
SN is assigned a sequence of intervals, which repeat until sensor lifetime ends. At the end of each interval, the sensor sends a fake message if it does not have an event to report. This should cause different delay times depending on the event relative arrival time and the length of the interval.

Considering the earlier example, we have ω*_max_* = 8, ω*_min_* = 2 and ω*_ave_* = 5. That is: we have reduced the delay interval by 37.5%. If m = 8, then delay reduced by 44%. The transmission of real and fake messages is exhibited in Figure 14.

#### 7.2.2. Reducing the Amount of Fake Messages and Delay for Real Messages

**Figure 15:**
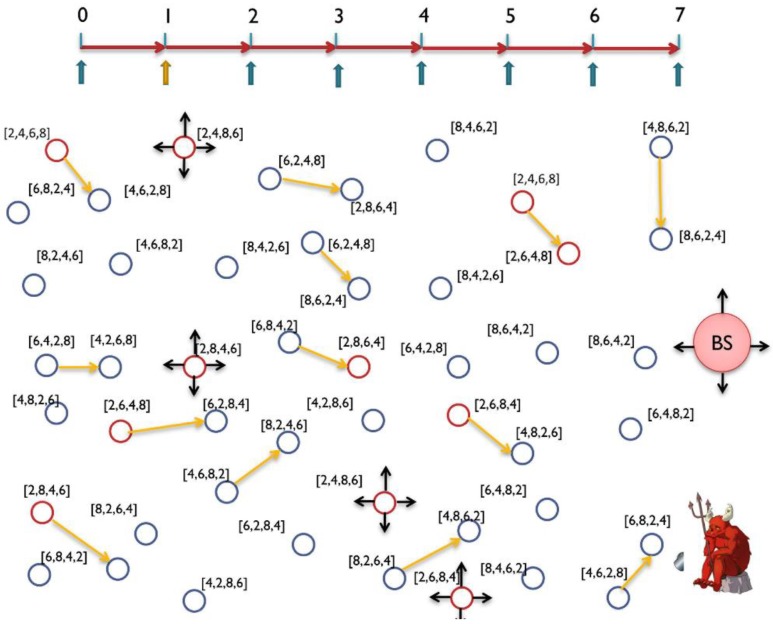
Any node assigned subinterval ω_i_ = 2 will send a fake message if it does not have a real message to report. All the nodes in the network can send real messages within any subinterval.

We have created a mechanism for dynamic interval allocation. Let us call $\bar{\omega }$ the *big interval* which has *subintervals* ω_i_. It still makes sense to send fake messages at the end of each interval. However, having the real message wait until the end of the interval time, as exhibited in Figure 15, is not commendable because it increases the delay at each node. Let us presume the current subinterval ω_i_ is the maximum, which is 8 time-units according to the example discussed earlier. Let us presume that the message was sensed at 2 time-units and it is ready to be sent at 4 time-units. Following the SGAT rules, it still needs to wait for another 4 time-units to be sent out! However, we know for sure that many other nodes have different ω_i_ subinterval values. Thus, during the time subintervals 2, 4, 6, and 8 there is enough traffic in the network. We propose that when the data is ready, the SN_i_ should send the data during the next subinterval slot within the current interval ω_i_. Consequently, if we are at interval ω*_max_* = 8 which has four subintervals at (2, 4, 6, and 8), and for our example, at 6 time-units the data can be sent out. This way, we save about 2 time-units delay while we can guarantee that the adversary will not be able to infer the source of transmission because we have enough traffic distributed in the network.

If we select higher values for $\bar{\omega }$, then we can further reduce the number of fake messages transmitting at one subinterval, however, we are increasing the average delay as well. Selecting a value for $\bar{\omega }$ could be a tool to adjust security *versus* energy conservation. We have improved the fake message efficacy (*FME*) [27,42] which could be calculated as indicated below:

(45) $$\text{FME}=\frac{\sum_{\text{i}=1}^{\text{i}=\text{m}}\frac{{\;}_{\bar{\text{ω}}}}{{\text{SI}}_{\text{i}}}-\text{m}}{\sum_{\text{i}=1}^{\text{i}=\text{m}}\frac{{\;}_{\bar{\text{ω}}}}{{\text{SI}}_{\text{i}}}}$$

where $\bar{\omega }$ is the big interval value, (SIi) the subinterval value, (m) the total number of subintervals. For example, if SN assigned a sequence $\omega_{i}\in [4,\;6,\;2,\;8]$, fake messages could be sent at the following subintervals [4, 10, 12, 20, 24, 30, 32, .....], and the real messages could be sent at the following sub intervals [2, 4, 6, 8, 10, 12, ....]. By substituting ω_i_ = 8, SI_i_ = 2 and m = 4, FME will be 60%. Figure 14 exhibits the transmission of fake and real messages for two consecutive subintervals.

#### 7.2.3. Energy Conservation by Forwarding Messages to Energy-Rich SNs

When a node senses data or receives data that needs to be forwarded to the BS, it has to select the next one-hop node from the neighborhood set N_i_. During the setup phase, each SN_i_ has information about the hop-distance for each of the neighbors stored in its table T_i_. Typically, there are three sets: one set where the hop-distance is less than its own (uplink set), a set where the hop-distance equals to itself (equal-link set) and a set where the hop-distance is larger than itself (downlink set). Choosing a node randomly or by round robin from the uplink set will be ideal in terms of delays since it will give the shortest path to the BS. However, that will cause the nodes in this set to consume more energy compared to the other two sets of the neighbors. After each transmission, the SN consumes some energy. The residual energy for SN_i_ will be calculated as below:

(46) $${\text{Δ}}_{residual}=\frac{{\text{Δ}}_{current}}{{\text{Δ}}_{initial}}$$

**Figure 16:**
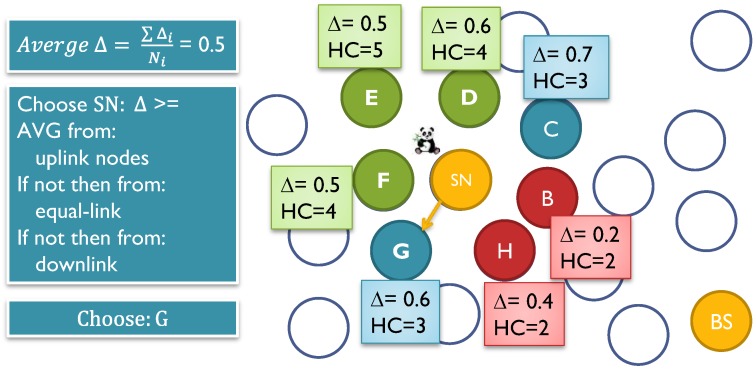
How to choose the forwarding node according to the energy levels of the neighbors. The sensor calculates the average energy levels for all the neighbors. Then it will select a neighbor, which has energy level higher than the calculated average energy, from uplink nodes if it is available. If not, then from equal-link nodes and then from downlink nodes.

Each node will calculate its residual energy and share it with its one-hop neighbors. When the node sends fake messages, it will send its residual energy with it. The neighbor SN_j_ will store the value in its T_j_ for each of its neighboring nodes. This way, any sensor node will have some information about the residual energy level for its immediate neighbors. Figure 16 exhibits the mechanism for selecting the forward node.

#### 7.2.4. Handling Rate Attack

**Figure 17:**
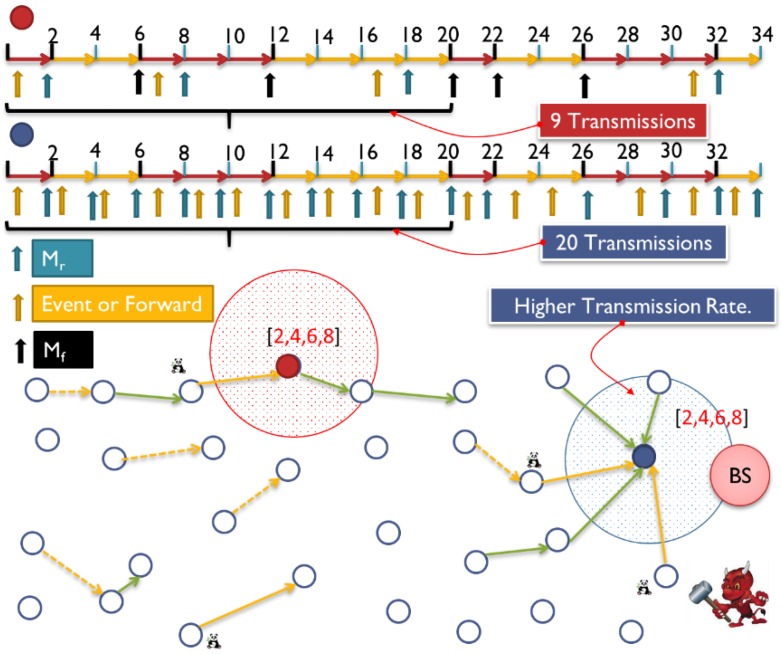
Higher transmission rate next to the BS. The figure exhibits about 20 transmissions near by the BS while one other area has 9 transmissions for the same period. This could be a bed for rate attack where the ADV can locate the BS [42].

**Figure 18:**
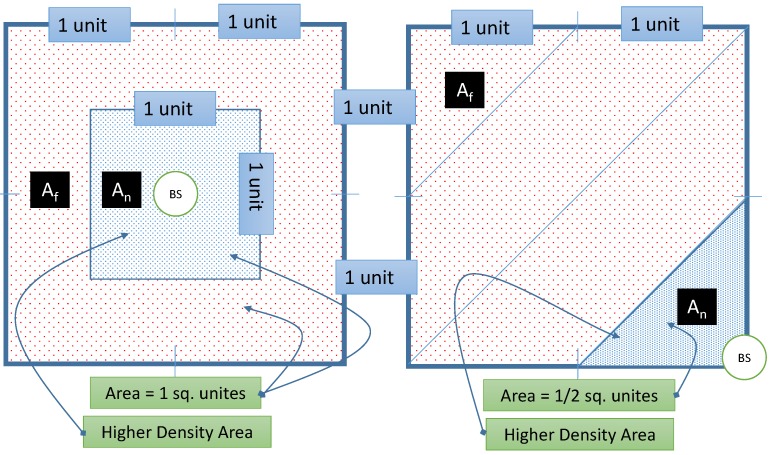
The area coverage of a central sink is higher than a peripherally sink. To balance the higher data rate nearby the sink, we acquire a higher density sensor distribution.

One issue that WSN with one sink could suffer from is having higher transmission rate next to the BS where messages ultimately need to reach out to the BS as the final destination. In contrast, periphery sensors far from the BS could have light transmission rates. Figure 17 illustrates the issue. This could jeopardize the location privacy of the BS. One solution is to have multiple sinks distributed in the network. This contradicts with the pre assumptions we set for our framework so we will not address this solution in this work. The framework needs to maintain similar average rate among all the sensors. This could be achieved by increasing the number of fake messages transmitted by less busy nodes, which means increasing the bandwidth usage and the power consumption. We need also to reduce the fake messages sent by busy nodes or delay the real messages to maintain similar rates. The latter is achieved automatically since the sensors do not send fake messages when they have real messages. However, this could be better tuned for average busy nodes as well. Having balanced rate in the WSN could help to maintain balanced average lifetime for the nodes in the network. Presuming that all the nodes are heterogeneous in terms of energy would mean that busy nodes would be depleted sooner that could create an empty coverage area or a buffer zone between the sink and the peripheral SNs. This makes it a double fold problem. The first approach is to select a suitable location for the BS in the network map. Most of the literature shows a side location for the BS. It is maybe because it is more suitable for the applications in hand where the BS is connected to the backbone network in a reachable area and sensors are unattended in out of reach areas. Figure 18 exhibits that the coverage area of a central BS is much better than a side BS. The density of nodes closer to the BS should be higher. The range of transmission for sensors in higher density areas may need adjustment to control energy dissipation. We could have multiple density areas around the BS where the density is reduced, as it gets distant from the BS. Figure 18 exhibits only two density areas for simplicity. If the storage of the sensor is not big enough, which is unrealistic case with increasing storage technology in the sensors, then the sensor does not need to include all the neighbors in the tables. The network will be divided into two areas, near (A_n_) and far (A_f_). The framework will set average transmission rate (ATR) thresholds, R_max_ and R_min_. Sensors in A_n_ will be loaded with R_max_ where the sensors need to queue messages to maintain the threshold. In reverse, sensors in A_f_ will be loaded with R_min_ to maintain the lower threshold by sending more fake messages as needed. The sensor will calculate its average transmission rate over a period of time T_atr,_ which is preset by the framework.

### 7.3. Contribution of Temporal and Rate Privacy Module

Our innovation in this module is by providing both temporal and rate privacy. Many works have provided solutions for temporal privacy by either using delays or fake messages, but few has addressed the rate privacy as an independent threat to the WSN. In our module, we have used delay and fake messages to provide an efficient solution for such attacks. We took in consideration, the need to reduce the delays in the real-time applications and the necessity to reduce the energy dissipation. In addition, very few works has addressed the rate privacy for the BS presuming it is physically protected. In this framework, we always considered the BS as a normal sensor node, which requires privacy. Section 9 provides a thorough analysis and simulation for the delays, entropy and energy. The three modules of anonymity, authentication, temporal/rate privacy altogether will provide source, link and sink location privacy.

## 8. Anonymity and Security Analysis

We need to analyze FAC for both passive and active adversary attacks. The adversary (ADV) model has a global view of the network. ADV could target the source, intermediary and BS nodes. Usually, ADV starts by monitoring transmission somewhere in the network and then attempts to acquire sources (downlink direction) or BS (uplink direction). Passive attack is ordinarily the base for active attack. Once ADV determines the identity and location of a source or the BS, it consequently can launch various active attacks against certain nodes or disrupt the operation of the entire WSN. The main strength of passive ADV is the fact that neither SNs nor the BS will know about their existence. Nonetheless, active attacks can be detected if the framework instruments reasonable IDS. Any comprehensive solution for location privacy should protect against anonymity attacks, temporal attacks and rate discovery attacks. We believe that routing privacy is useful only against local advisory and once the WSN faced with a global or a multi-local adversary, routing privacy is not crucial. Thus, we have chosen short-path routing technique for this work. Any other routing protocols should be utilized to reduce delays and energy consumption.

### 8.1. Security against Passive Attacks

SNs use disposable pseudonyms to identify each other instead of using real IDs. No real ID stored in the sensor and no pseudonym is used more than once. Data is encrypted all the way from the source to the BS using shared pair-wise keys. For *eavesdropping and content analysis*, ADV can intercept messages without being able to read them because data is encrypted all the way to the BS. The only information ADV can get from the captured messages is the pseudonyms: OHPID, BPID or FPID, which are all temporary IDs and have no use except to calculate a new set of pseudonyms. Fortunately, the ADV cannot get from the captured messages, important parameters a_i↔j_,b_i_ or c_i_ which are all required to calculated new pseudonyms. Source PIDs are all encrypted during transmission. For *hop-by-hop trace*, ADV can track a stream of messages from one node to another by overhearing the messages. The ADV will be challenged with many real and fake transmissions throughout the WSN. Furthermore, each node will retransmit the messages through different routes. For *size-correlation*, ADV will be able to understand relationship between incoming and outgoing messages by analyzing sizes of the messages. This attack does not work for our framework since all the messages have commensurate size. For *identity correlation*, ADV cannot relate overheard identities to their nodes. It is not possible since SNs use different pseudonyms every time a message is transmitted. For *rate monitoring*, ADV tries to collect some statistical information about transmission rates. For instance, WSN will have a higher transmission rate nearby the sink. This is handled by issuing fake messages to maintain a similar transmission rate. For *angle-of-arrival (AoA)*, ADV uses special hardware to determine the signal direction. The framework did not account for specific countermeasure; however, it becomes a more serious issue with mobile SNs. Furthermore, *AoA* would not perform well in our framework because of the uniform message distribution by using real and fake messages. For *received-signal-strength (RSS)*, ADV uses special hardware to measure signal strength to calculate distance to the source. This is not an issue for our framework since every transmitter has fixed transmission power and SNs are immobile.

### 8.2. Security against Active Attacks

In principle, we assume ADV knows encryption protocols used by the framework; however, the framework needs to hide *encryption keys* and *IDs*. Active attacks can be categorized into *soft* and *hard*. For soft-active attacks, ADV tries to compromise SNs to get some information related to security of the sensors such as *keys* and *IDs*. Consequently, it will monitor all messages traversing through the compromised nodes to discover the source and the BS locations. ADV hides its presence by acting passively (soft) but once it captures privacy information, it reports the information to an external executer to do further damages (such as killing the Panda in the Panda game). For that, it is harder for the IDS to detect the attack. In hard-active attacks, ADV captures SNs and invasively forge messages, sent replay messages *etc*. Moreover, ADV could load powerful devices with the captured credentials to launch more catastrophic attacks. Hard-active attacks could be detected by IDS; however, it could depend very much on the sophistication of the IDS used. With that, it remains very challenging to countermeasure hard-active attacks. In the following two subsections, we will analyze the security of our framework against active attacks.

#### 8.2.1. Soft-Active Attacks

If ADV *physically* compromises SN_i_, then it captures two sets of information:

(i) Information related to the node itself: the current *PID*, the parameters used to calculate the pseudonyms, the hash functions, the keys and other information as listed in Table 2.
(ii) Information related to the neighbors as listed in Table 3.

The ADV would have all it needs to issue new valid pseudonyms and to send messages out to neighbors. Let us look closely at few scenarios:

*Scenario 1*: If ADV physically compromises SN_i_, and if SN_j_ and SN_r_ ϵ N_i_, so SN_i_ knows some information about both SN_j_ and SN_r_. However, it cannot calculate important information such as a_j↔r_ which is required for one-hop communication between SN_j_ and SN_r_ [16], because SN_i_ would need ID_j_ and ID_r_ which are both deleted permanently at the end of the setup phase. If SN_i_ hears a message, it cannot determine, with high confidence, the sender among neighbors while communicating with each other. If SN_i_ receives message from sources ∉ N_i_, then it would not be able to determine the source.

*Scenario 2*: If ADV *physically* compromises multiple SNs, let us call it set N_cs_, and collects number of messages, let us call it set N_cm_. Then, the number of compromised PIDs equal to N_cm_ since each message has unique PID. If the source SN_i_∉ N_cs_, then ADV cannot know the source node [16,27].

*Scenario 3*: If the message sent, by source SN_i_ as in *scenario*, 2 passes thought SN_j_ ϵ N_cs_ or even through multiple compromised nodes, it will not be able to correlate the captured PID_i_ with SN_i_. 

*Scenario 4*: If a message sent by source SN_i_ and ∀ SN ϵ N_i_ is also ϵ N_cs_ (all neighbors are compromised), then ADV will be able to know that SN_i_ is the source. It is unrealistic situation to have many compromised nodes in one area. However, this proves single or few compromised nodes cannot locate the identity of the source. In addition, a compromised node does not actually need to locate the sources within its range since it can detect the objects of interest (Panda) knowing that the ultimate goal of the adversary is to capture the *object* not the sensor reporting the object.

In summary, while we cannot prevent physical capturing of sensors, we need to make sure capturing sensors do not have destructive effects on other sensors. It is clear that our anonymity model protects against the *avalanche* or the *domino effect* behavior once one or few sensors are physically captured.

#### 8.2.2. Hard-Active Attacks

If ADV physically compromises SN_i_ then it can launch denial of service attacks (DoS), which is an effort to temporarily or indefinitely suspend transmission in the network. It consumes the resources such as bandwidth, memory, storage, and processor time. When ADV compromises SNs, it would be able to send massive valid messages to consume system resources. The ADV will be able also to launch replay attacks where ADV gets credentials of the some sensors and attempts to mimic the sensors to send messages to other neighbors. The other attacks such as, forging attack, packet alternation, packet dropping and packet injection are all only possible to physically captured nodes. However, it cannot propagate easily behind neighbors. Nothing could be worse than having physically captured nodes where ADV has full control over the sensors. Good IDS can detect such attacks and respond by removing the compromised nodes immediately. The most danger tactic of hard-active attacks is to prevent the real messages from following normal paths to the BS and force the messages to traverse through certain routes. Our main contribution to handle this attack is to put in place a seamless and efficient protocol to add and remove SNs while WSN in action.

### 8.3. Sink Security

**Figure 19:**
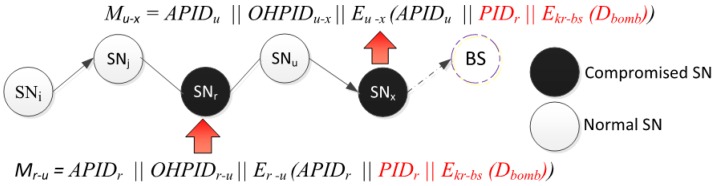
Hard-active Attack tries to get the BS by inserting a signature in the transmitted message.

ADV can learn that a sensor has received a message in two ways: (i) When the sensor retransmits the message, which was tracked beforehand to another sensor; (ii) the ADV is able to make a correlation between the captured ID and the physical recipient sensor. The adversary cannot locate the BS location by compromising only one neighboring sensor because each transmission uses a different pseudonym. It actually will need to compromise multiple colluding sensors along the path to the BS or many neighbors of the BS. While we cannot prevent having many physically fallen sensors, our framework’s goal is to delay the capturing of the BS if there are many colluding captured sensors in the WSN. A very interesting *scenario* is *exhibited* in Figure 19. Let us presume SN_r_ ϵ N_cs_. It issues a message with D_bomb_ such that: *APID_r_ || OHPID_r↔u_ || E_r→u_ (APID_r_ || PID_r_ || E_kr↔bs_ (D_bomb_))*. IF ADV compromise multiple nodes along the path to the BS where each sensor decrypts the data to read this *signature* at every hop: *(PID_r_ || E_kr↔bs_ (D_bomb_).* Providing the colluding sensors, in the path to the BS, read similar signature while it knows by design that every message should be directed uplink to the BS, the ADV could follow through to the BS. Having multiple compromised paths (with compromised sensors) reading the same pattern will give adversary more clues. Compromised nodes can even collude to force the real messages to route through fixed suspected areas in effort to focus the capturing process, which becomes a function of: (i) the size of the network; (ii) The traffic density; (iii) the number of compromised nodes. To solve this issue, we have to wipe out the signature before each transmission. Thus, every message will be forwarded to the next hop as below:

M_u→x_ = APID_u_ || OHPID_u↔x_ || E_u→x_ (APID_u_ || PID_r_ || **PID_u_** || **E**_ku↔bs_ (E_kr↔bs_ (D_i_))) (47)

We have added a multiple levels of encryption, which will be done at every hop using the shared key between the hop and the BS. In addition, PID of the hop will be added in sequence so the BS can do the decryption in sequence. This solution increases the size of the message proportionally to the number of hops. We suggest having the onion encryption done for a distance of few hops, O_h_. So, if O_h_ = 2, then we have only two extra encryptions. In addition, we need to account for O_h_ PID’s added to the message.

We have implemented using Matlab a WSN of 100 SNs uniformly distributed over 30 × 30 area where the average distance between the SNs 3.7 as in Figure 12. The nodes are homogeneous in terms of initial energy. The WSN adopted one BS located at the side of the network. The SNs were preloaded with all the initial pseudonyms, so the simulation started right at the communication phase.

**Figure 20:**
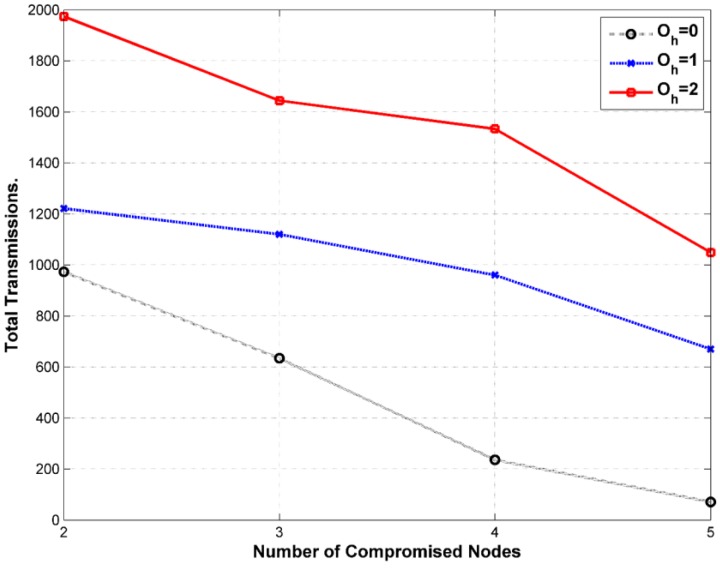
Protecting the BS by having onion encryption. Increasing O_h_ and decreasing the number of compromised nodes will increase the total number of messages successfully transmitted to the BS before it is captured.

Sensors issue real messages according to a normal distribution using SGAT. To simulate how the network behaves to protect the BS, the simulation inserted some random compromised sensors. The compromised sensors sent some bomb messages as exhibited in Figure 19 and colluded to track the location of the BS. We have protected the BS by using the onion encryption so, we have simulated for O_h_ equals to 1, 2 and 3. The adversary succeeds when it identifiers all the nodes forming the curve around the BS; SNs have 1, 2 and 3 hc from the BS, consecutively. Figure 20 exhibits the number of transmissions required before the adversary can succeed. It is clear that with higher value for O_h_, the network will be able to send more messages before the BS is compromised. Having a higher number of compromised nodes in the WSN will make it faster to capture the BS, as well.

### 8.4. Link Anonymity

Link anonymity is to prevent the ADV from knowing the relationship between the sender and the receiver. If a message leaves a sender and subsequently leaves the recipient without change, the ADV would know the relationship between the two nodes. This is secured in our framework since every message is completely changed after each retransmission including the IDs. In addition, it maintains fixed size. Applying different delays and different next-hop direction should also increase the privacy of the link. Furthermore, the adversary cannot know if the link carries real or fake data.

### 8.5. Timing Privacy

By using fake messages at variable interval times and message delays, it becomes super hard for the ADV to correlated messages being transmitted over the network as exhibited in Figure 12.

### 8.6. Routing Privacy

Although short-path routing is used in this framework, choosing the next hop is done according to certain *probabilistic* algorithm, which accounts for the residual energy of the sensors and the usage frequency to increase the route privacy, as exhibited in Figure 16. ADV cannot relate routes to nodes due to the triple anonymity. Even if two messages for one sensor follow the exact same route, ADV will see them as if they are two different routes since each hop along the route carries messages with different PIDs.

### 8.7. Data Privacy

All the data is encrypted before transmission and encrypted again at every hop along the route to the BS. A message digest will authenticate data. The only time data is not protected when the sensors are physically compromised. The compromised nodes are able to inject data in the network. If ADV uses the compromised nodes actively, a good IDS can detect the falsified data. The framework provides a secure facility to remove compromised sensors and to add valid sensors, if needed, to the WSN.

### 8.8. SLP and BLP

SLP and BLP are achieved at first by having the triple anonymity (*source*, *BS*, *link*) which was argued earlier. ADV cannot infer any information from the intercepted messages. Passive attacks will not endanger the location privacy. However, strong active attacks could hinder the location privacy without having good IDS. Secondly, we have provided a solution for temporal privacy using ECAT. Thirdly, we have provided a solution for rate attacks. The three security measures will work hand in hand to provide location privacy.

## 9. Performance Evaluation

In this section, we evaluate the performance of the FAC framework, including delays, energy dissipation, data rate privacy, storage, processing, computational, and communication costs.

### 9.1. Delay

**Figure 21:**
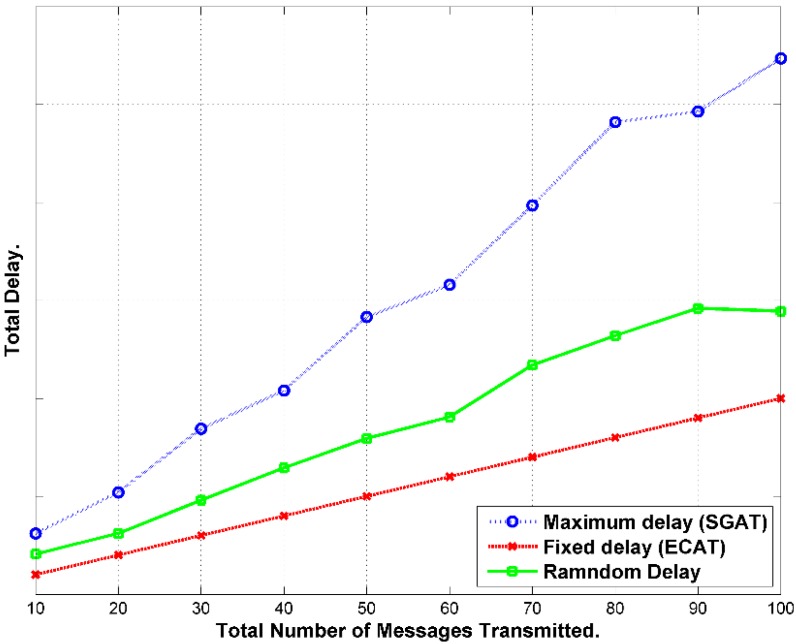
Total accumulated delay per one node increases as number of messages increases in the WSN. ECAT sends the message only one subinterval after the message arrival. SGAT sends the message at the end of the big interval $\bar{\omega }$. In between, the approach of selecting one of the following subinterval randomly to send the message. Clearer image?

**Figure 22:**
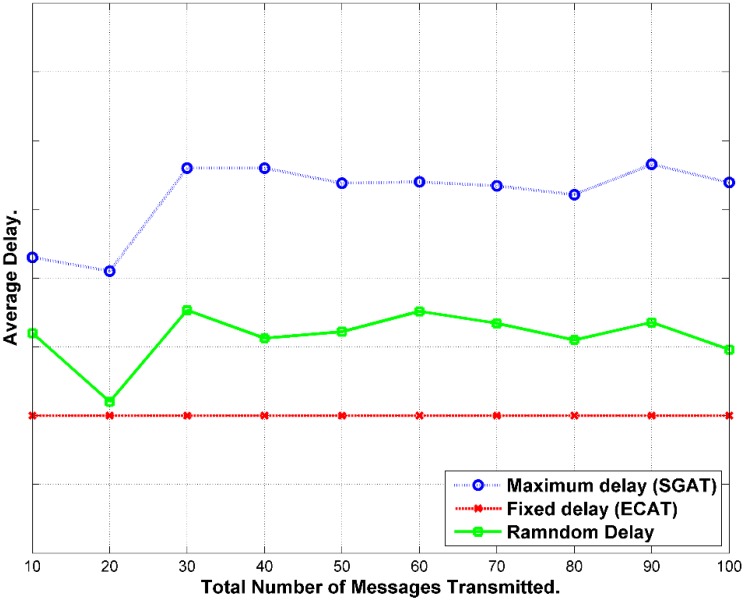
Average accumulated delay per one node. ECAT shows the good performance of a minimum average delay since it sends the message only one subinterval after the message arrival.

**Figure 23:**
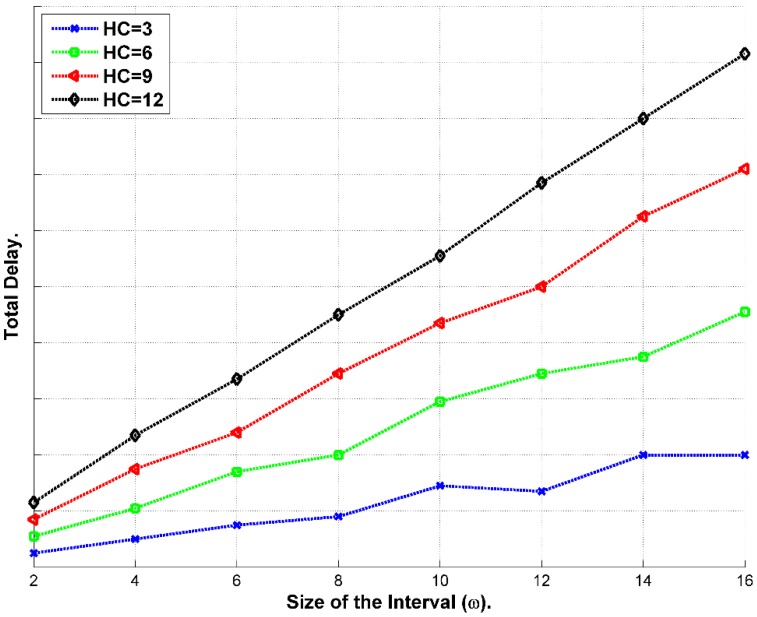
The accumulated delay is a function of the hop count (hc) and the size of ω.

In SGAT, sensors transmit/forward the data at the end of the interval, which would cause a huge delay considering the volume of messages that each sensor needs to transmit during the network real-time operation. In addition, the messages traverse through multiple hops until it gets to the BS, which makes the accumulated delays significant. The other alternative scheme is having the sensors select one of the following subintervals (ω) randomly to forward the message. This also will cause some unnecessary delays although it could help in hiding the temporal behavior of the sensors. ECAT scheme divides $\left(\bar{\text{ω}}\right)$ into subintervals (ω), so the transmission will happen at the first available subinterval when the message is ready. We have simulated a smaller network to the one descripted in Section 8.3 for the transmission delays. It includes 48 SNs only with ω distribution as presented in Figure 15. We have three simulations using SGAT, ECAT, and random delays. Figure 21 shows that delay per one-transmission increases throughout the network as the number of transmitted messages increases which could cause unjustifiable delays especially in the real time applications. Figure 22 also shows the average delays for the three schemes. It shows that using ECAT has improved delays by 64% compared to SGAT. The total delay for one message from a source to a destination (BS) is calculated according to expression Equation (37). It is a function of the distance from the BS (hc) which we technically have no control over after sensors deployment. In addition, it is a function of the chosen ($\bar{\omega }$) and (ω) values for the system. The larger the (ω), the more delays accumulated. We have simulated the same network using ECAT for the total delay as exhibited in Figure 23. It shows that the delay rises as the *hc* increases and as the size of the intervals widens. We conclude of these simulations that the performance of ECAT is better than SGAT while it continues to provide a good temporal privacy. Using a fixed delay will reduces the delays slightly but it provides a very week temporal privacy.

### 9.2. Energy Cost

In our work, we will assume a simple energy dissipation model [27,42,48,49]. The radio dissipates Ԑ nJ/bit for both transmission and reception by the sensors circuitry. Moreover, it consumes ԑ nJ/bit/m^2^ for the transmitter amplifier to achieve an acceptable signal to noise ratio. Therefore, to transmit *k* bits for *r* distance, the total transmission energy dissipation will be:

(48) $${\text{E}}_{\text{t}}=\text{k }\times \;Ԑ+\text{k}\times {\text{ r}}^{2}\times \text{ε}$$

In addition, the receiver would consume for reception of k-bit message:

(49) $${\text{E}}_{\text{r}}=\text{k}\times \;\text{Ԑ}$$

SGAT assumes that every node would send one message at the end of each interval where the message could be either real or fake. If we have *N* nodes in the network, then we expect *N* messages during each interval. The energy spent for transmission or reception is similar per one message because we have unified-size messages to prevent size correlation attacks by the adversary. If we have *p* percent of the nodes issue or forward real data at each interval, then *1 − p* percent of the energy and the bandwidth is wasted on fake messages. We can adjust the amount of energy consumed by increasing the interval time (ω). However, increasing (ω), would increase the delay. The consumption of transmitting fake messages is a double fold since the transmitter will consume ${\text{E}}_{\text{t}}$ for every message and all the neighbors (*Ni*) will consume $(N_{i}\times E_{r}$). When the transmission range increases, *N_i_* increases. The total energy consumed in the network to send real and fake messages in one interval [48]:

(50) $${\text{E}}_{\text{R}}=\left(\text{N}\right)\left(\text{k}\;\times \;\text{Ԑ}+\text{k }\times {\text{ r}}^{2}\;\times \text{ ε}\right)+\left(\text{N }\times \;N_{i}\right)(\text{k}\;\times \;\text{Ԑ})$$

**Figure 24:**
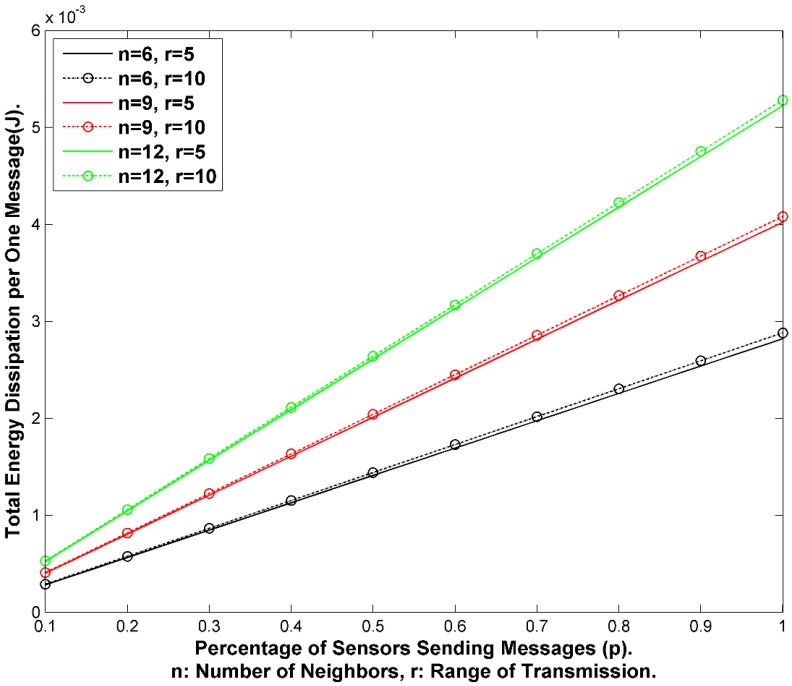
The energy dissipation increases as the number of neighbors and the sensor transmission range increases. Simulation assumed message size of 8000 bits, Ԑ is 50 nJ/bit and ԑ is 100 pJ/bit/m^2^.

**Figure 25:**
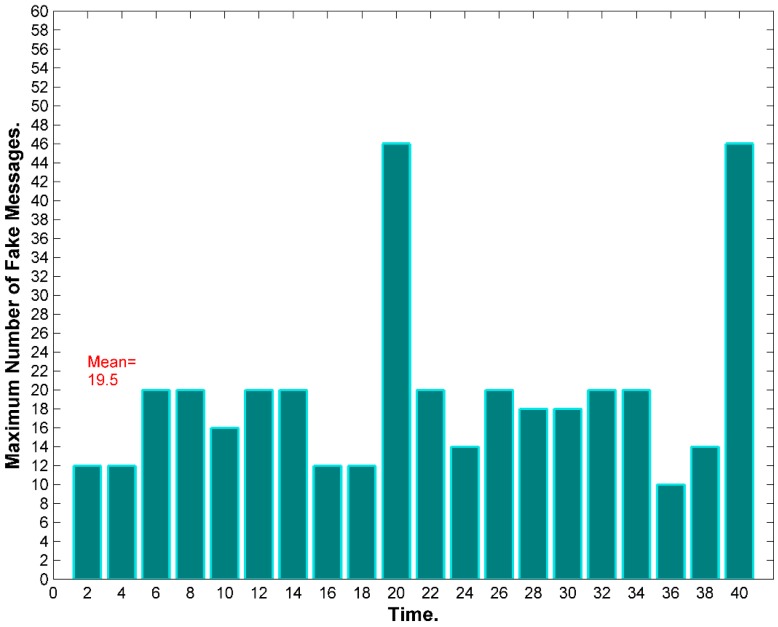
A simulation for the maximum possible fake messages per subinterval using ECAT. In SGAT, this number should be 48, which is one message per one sensor. ECAT has reduced it significantly. The mean in this simulation is 19.5, which is about 59% reduction of fake messages.

ECAT has improved the energy dissipation by reducing the amount of fake messages transmitted while maintaining the required temporal security. The number of total messages transmitted per interval has reduced from 100% to a certain percentage (*p*). We have simulated the WSN in Figure 15 as presented in Section 9.1 using SCAT to calculate the energy dissipation. Figure 24 exhibits the total energy dissipation per one message considering the transmitter, the recipients and the range of transmission. The size of the messages is 8000 bits, Ԑ is 50 nJ/bit and ԑ is 100 pJ/bit/m^2^. The simulation shows that the energy dissipation due to the increase of sensor range is marginal compared to the increase in energy dissipation due to the increase of neighbors (*N_i_*). However, increasing the range could increase N_i_ if the WSN has uniform sensor distribution. We have also simulated the network to see how the transmission of fake messages has improved using ECAT. Figure 25 exhibits the simulation of 40 subintervals (ω). The graph shows the maximum possible fake message at each subinterval (ω). For instance, the total fake messages during ω = 10 is 16 messages while during ω = 32 is 20 messages. The mean of fake transmissions is 19.5 (compared to 48 messages in SGAT). The average fake messages for the completely simulated period is 19.5 messages which shows about 59% reduction of possible fake messages comparted to SGAT.

**Figure 26:**
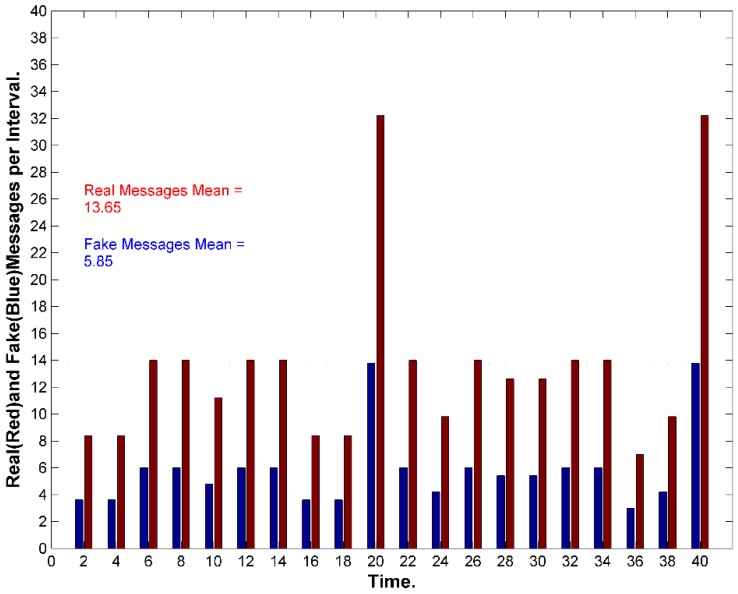
The average fake messages in a busy network with 70% of the slots occupied by real messages. The reduction of fake messages transmission in ECAT is about 88% compared to SGAT.

The number of fake message will be reduced further as the network gets busy transmitting real messages since a sensor node do not send a fake message at a subinterval where it has a real message to convey. We have simulated the same network with 70% probability of event occurrence. Figure 26 shows that the average fake messages has reduced to 5.85 messages, which is almost 88% reduction from SGAT. This also will reduce the energy consumption significantly.

The most expensive operation for energy consumption is transmission of *bits* from one node to another. We use two stages for air communication in our framework, (i) In the setup phase and; (ii) in the communication phase. The data transmission during setup phase is minimal. During communication phase, data will be forwarded hop-by-hop to the BS. Every packet is equally sized to prevent time and size correlation. We have introduced a probabilistic fake message transmission scheme which none of the other protocols adopted. Real messages are sent at the end of each subinterval time to prevent delays.

The cost per message at one interval time is:

(51) $$\text{Average}\;\text{Message}\;\text{Cost}=\;\frac{\text{R}+\left(\text{N}-\text{R}\right){\text{P}}_{\text{r}}+\text{A}}{\text{R}}$$

where *R* is the total number of SNs sending real messages at one subinterval time, *P_r_* the probability of sending fake message by SNs, and A is the average number of acknowledgements in one interval. None of the other schemes addressed the issue of rate analysis attacks, which is one of the easiest attacks any adversary can use. Using fake messages is an expensive solution. However, we have designed FAC to be adaptive to the network traffic situation by using a closed-loop system. The sink can always increase or decrease the amount of fake messages used according to the reports it is getting about the system security. The threshold values of R_min_ and R_max_ are also adjustable according to the network situation.

### 9.3. Transmission Rate Privacy

**Figure 27:**
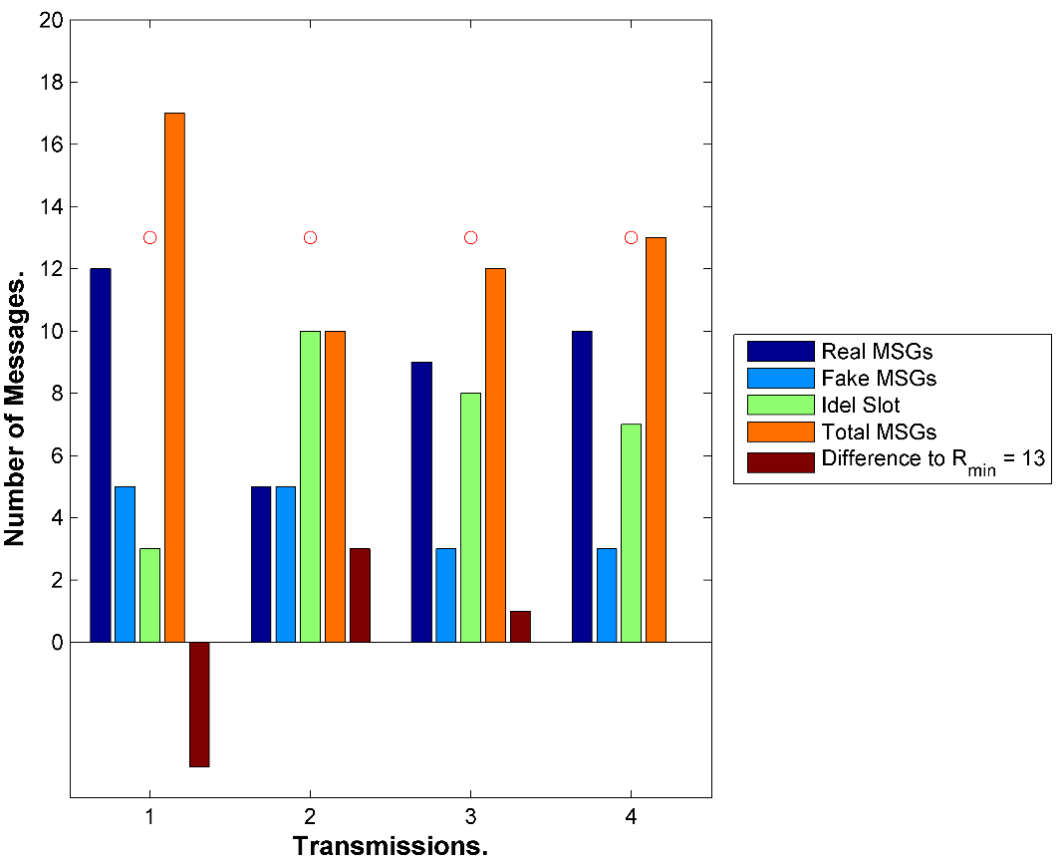
The simulation shows the total real messages, fake messages and idle slots. The threshold rate is *thirteen* messages over *T_atr_* period. For instance, the first transmission shows total of *sixteen* messages, which is *three* above the threshold value. The sensor will cancel three fake messages. The third set shows the number of transmissions at the threshold.

To handle this issue, we have adopted two threshold values: Rmin and Rmax where the sensor needs to keep its message transmission rate between these two values. The sensor needs to send real message at the end of subinterval time slot. If it does not have a real message, then it needs to send a fake message only if that time slot is scheduled to send a fake message according to ECAT protocol, otherwise no transmission will happen and the slot remains idle. Ideally, the sensor has a real message at the subinterval so it does not need to waste a slot by sending a fake message. The sensor can use this facility to control the threshold data rate. For instance, if the rate is high (such as in areas nearby the BS), it can replace fake messages with real messages which is a double fold beneficial. If all the fake messages are already replaced and still there is real messages above the threshold, then the sensor is required to queue the messages and delay the transmission to maintain same average message rate between *Rmin* and *Rmax*. In contrast, if the message rate is low (as in the periphery sensors), then the sensor can transmit more fake messages during idle slots. We have simulated the network in Figure 15 for ECAT and assigned the *Rmin* to be *thirteen* messages for two consecutive $\left(\bar{\omega }\right)$ intervals (a total of 20 subintervals). We have assigned the Rmax to be 13 messages during this period, which is seven less than the total number of subintervals. That means we allow up to 13 real and fake messages during these two consecutive $\left(\bar{\omega }\right)$ intervals. Figure 27 exhibits the output of the simulation for four different individual transmissions. For instance, the first transmission shows, 14 real messages (blue bar), 2 fake messages (light blue bar), and *four* idle slots (green bar). The total real and fake messages is 16 (*orange bar*) which is above the assigned threshold, *thirteen*, by *three* messages, which is expressed by the brown bar.

The SN will cancel *three* fake messages out of the *four*. In some worse cases, the sensors would need to queue the messages for next slots. For instance, if there is 15 real messages during this time, the system send only 13 messages and queue 2 messages for the next period. Ultimately, the overall message rate during T_atr_ will be within the assigned thresholds.

**Figure 28:**
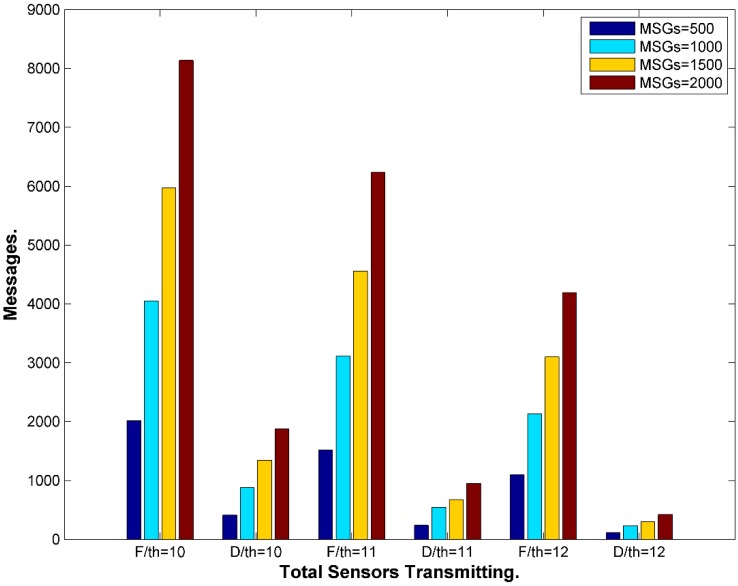
Simulation for busy network with different R*_min_* threshold values th = 10, 11 and 12. F stands for the number of fake messages has been reduced and D stand for the number of delayed messages to maintain R_min_. The simulation shows that the number of delayed messages is minimal while it decreases as the threshold increases.

We have simulated this approach extensively for (500), (1000), (1500) and (2000) transmissions as exhibited in Figure 28. The first, third and fifth bar sets show the total amount of fake messages needed to be replaced with real messages to maintain *R_min_* for the thresholds of th = 10, th = 11 and th = 12 consecutively. For instance, a threshold of 10 means that the maximum number of messages transmitted should be 10 (out of 20 in our simulation). The second, fourth and sixth bar sets also show the number of messages which needed to be queued for the three consecutive threshold values. Therefore, if we have real messages above the number of scheduled fake messages, then we have to queue the messages for the next period of T_atr_. Overall, this simulation exhibits a great preference since we always would like to reduce the amount of fake messages and keep the bandwidth busy with real messages whenever it is possible. In addition, the simulation exhibits very small messages need to be queued (delayed). It shows as we increase the threshold value the less fake messages replacement or delays is required. In summation, reducing the fake messages and keeping the delayed message minimal is the goal, which ECAT clearly achieves.

### 9.4. Storage Evaluation

There are two sets of information stored in a SN_i_: (i) information related to the sensor itself such as random numbers: (a_i_, b_i_, c_i_), pseudonyms: (PID_i_, BPID_i_, FPID_i_, APID_i_), keys: (ki↔bs, bki, fbki); (ii) information related to neighbors which include, random numbers: (a_i↔j_, b_j_, c_j_), pseudonyms: (OHPID_i↔j_, BPID_j_, FPID_j_), keys: (k_i↔j_), Misc: (link_i↔j_, Δ_j_).

If we presume that the keys, the random numbers, the pseudonyms and the hash functions are all *n* bits long in average, and the required bits for miscellaneous data altogether is *two* bytes, and the average number of neighbors N_ave_, then the total storage memory required is:

Storage = 10*n* + (7*n* + 16) × N_ave_ (52)

Chen *et al.* [16] indicated the storage for *SAS*, CAS, *APR*, *DCARPS and EAC.* We also calculated the storage for *PhID*, *ACS*, HIR and RHIR. All are listed in Table 4. The size of storage increases proportionally when the size of *n* increases. The most common hashing functions which are considered very secure are: *MD4* [50] which uses 128-bits digest, *SHA-1* [50] which uses 160-bits digest, and *Whirlpool* [50] which uses 512-bits digest [50].

**Table 4:** Performance Comparison. *N* is the total number of sensors; *N_ave_* is the average number of neighbors; *k* (only for RHIR) is number of stored hash values where the SN stores *k* hash values per one neighbor which are calculated in advance at setup phase.

No.	Scheme	Storage Cost (bits)	Computation Cost
1	SAS	2nN + 4nN_ave_ + 16	No hashing operations
2	CAS	6n + 7nN_ave_ + 16	Two hashing orations and two encryptions
3	HIR	2n + 2nN_ave_	One hashing function
4	RHIR	2n + 2nN_ave_ + nkN_ave_	No hashing functions
5	APR	9n + 7nN_ave_ + 2N − 2N_ave_ − 2	Six hashing functions
6	DCARPS	3n	No hashing functions
7	ACS	5nN_ave_	Two hashing functions
8	PhID	(3n + 2) × N_ave_	Four hashing function
9	EAC	6n + 6nN_ave_ + 2	Four hashing operations
10	FAC	10n + (7n + 16) × N_ave_	Four hashing operations & O_h_ encryptions

### 9.5. Processing and Computational Evaluation

**Figure 29:**
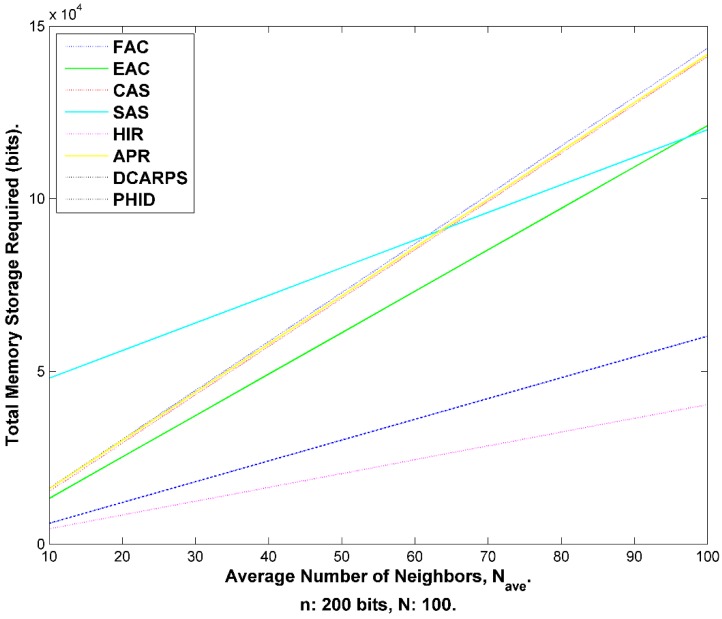
Size of storage memory using different privacy schemes.

Hash functions are used to calculate the pseudonyms and symmetric cryptography is used to encrypt the messages. Because we need to calculate three pseudonyms and one acknowledgement pseudonym after each transmission, using encryption to create pseudonyms was avoided since it requires more processing power compared to hash functions. When a SN senses data, it needs to calculate four OWH for PID, OHPID, and APID at the sender and OHPID at the receiver. If the system opts for data authentication, then another hash function is needed. The source node needs only one encryption for the data if O_h_ = 0, however, it needs O_h_ more encryptions if onion fashion is used. Each intermediary node needs one decryption operation and then another encryption to issue the new message. Chen *et al.* [16] indicates that SAS does not use hashing or encryption to create pseudonyms because it uses already created pseudonyms from a space. The other scheme by Chen *et al.* [16], CAS, uses two hashing operations and two encryption operations. APR uses at least six hashing functions. DCARPS uses constant IDs, so no hashing functions or encryptions for creating IDs. EAC has four hashing operations. It is clear that our framework needs a bit extra processing power due to the higher privacy and security we have achieved. None of the other schemes can achieve privacy against global threats and active adversary attacks. The power consumption due to the additional encryption operations is marginal compared to the power consumption caused by data transmission. Figure 29 exhibits different storage size for different privacy schemes, which are discussed throughout this work. It shows that the increase in storage is *linear* and relatively comparable to the other protocols. The size of the storage would increase when the number of neighbors increases. Each SN has limited flash memory size, which could confine the maximum number of neighbors that a sensor can fit. As an example, *TelosB* mote [16,20] has 1 MB external flash memory. Thus, if one neighbor node requires 1.2 k bits of storage memory, then TelosB could fit more than 800 neighbors, which is very much more than what is needed in practical networks. Although FAC shows a bit of increase in the storage required to store the pseudonyms but it is the only one, among the discussed protocols in this work, provides a steady and functional anonymity and location privacy under strong global and active attack. In addition, the current technology provides sensors with sizable storage memory, which makes it not an issue at all.

## 10. Conclusions and Future Work

FAC is a modular framework that provides source, link and sink anonymity. It also provides temporal privacy and rate privacy. None of the previous related-work have a comprehensive solution for anonymity and location privacy. The three modules provided in FAC are made work together to prevent any statistical analysis attacks. The quadruple privacy (anonymity, temporal, rate, statistical) has provided a fortified SLP and BLP. FAC has addressed both local and global adversary. We have used a complex anonymity module where pseudonyms to replace real IDs are used. To provide temporal privacy both delays and fake messages are used. The use of fake messages was adjusted to manage the energy consumption. Two schemes are introduced, SGAT and ECAT. FAC is able to handle both homogenous and heterogeneous sensor nodes. FAC is both energy-aware and delay-aware. We have demonstrated that FAC can withstand passive and active attacks by presenting scenarios and provided solutions. The memory cost was mathematically analyzed for the framework. The computational complexity for encryptions and hash functions was analyzed. To provide security for the BS against colluding active attacks, we have introduced onion encryptions. We have simulated the performance of the framework. The future work would include enhancement on the fake messages probabilistic scheme. In addition, we will implement FAC for different routing protocols such as clustered networks. We would plug FAC in some civil and military applications for further analysis, development and improvement.
